# Quantitative analysis of the morphing wing mechanism of raptors: Analysis methods, folding motions, and bionic design of *Falco Peregrinus*

**DOI:** 10.1016/j.fmre.2022.03.023

**Published:** 2022-04-29

**Authors:** Di Tang, Xipeng Huang, Jinqi Che, Weijie Jin, Yahui Cui, Yangjun Chen, Yuxiao Yuan, Zhongyong Fan, Weiwei Lu, Siyu Wang, Yin Yang, Dawei Liu

**Affiliations:** a College of Mechanical Engineering, Zhejiang University of Technology, Hangzhou 310014, China; b Affiliated HangZhou XiXi hospital, Zhejiang University School of Medicine, Hangzhou 310023, China; c Zhejiang Museum of Natural History, Hangzhou 310014, China; d High Speed Aerodynamic Institute, China Aerodynamics Research and Development Center, Mianyang 621000, China

**Keywords:** *Falco Peregrinus*, Raptor, Suspension system, CT scan, Bionic wings, Four-bar mechanism

## Abstract

Raptors can change the shape and area of their wings to an exceptional degree in a fast and efficient manner, surpassing other birds, insects, or bats. Some researchers have focused on the functional properties of muscle skeletons, mechanics, and flapping robot design. However, the wing motion of the birds of prey has not been measured quantitatively, and synthetic bionic wings with morphing abilities similar to raptors are far from reality. Therefore, in the current study, a 3D suspension system for holding bird carcasses was designed and fabricated to fasten the wings of *Falco Peregrinus* with a series of morphing postures. Subsequently, the wing skeleton of the falcon was scanned during extending motions using the computed tomography (CT) approach to obtain three consecutive poses. Subsequently, the skeleton was reconstructed to identify the contribution of the forelimb bones to the extending/folding motions. Inspired by these findings, we propose a simple mechanical model with four bones to form a wing-morphing mechanism using the proposed pose optimisation method. Finally, a bionic wing mechanism was implemented to imitate the motion of the falcon wing—divided into inner and outer wings with folding and twisting motions. The results show that the proposed four-bar mechanism can track bone motion paths with high fidelity.

## Graphical abstract

**Figure:**
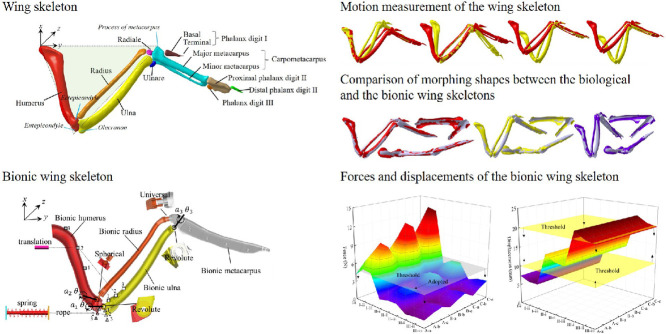

## 1 Introduction

Flying birds in nature can use flapping wings to gracefully and dexterously traverse terrestrial, aerial, and aquatic environments [1]. As concluded in our previous research, the key to high manoeuvrability in bird flight lies not in the static aerodynamic performances but in how the wing morphs to change the flying situations [2,3]. However, no aircraft has yet been designed to achieve morphing abilities of birds. Therefore, more attention should be paid to the myology and anatomy of bird wings, as long as they have a bionic design.

Wing morphing, the key to manoeuvrability flight, broadens the excellent performance of birds, enabling them to fly faster and adjust their attitude more effectively [4], [5], [6]. Therefore, the flying robot design inspired by flying birds has become a popular topic in mechanical engineering. An Eagle simulator model, with a weight of 425 g and a wingspan of 107 cm, was designed to study and verify relevant aerodynamic parameters during flight [7]. Gerdes et al. [8] fabricated the pioneering flight, ‘Robo Raven’, which had a wingspan of 150 cm and a weight of 690 g. A bird-inspired platform was studied to verify that independent wing control can provide a greater flight envelope. A modern flapping wing air vehicle, ‘SmartBird’, was developed by the Festo company. It is driven by two servo motors, with the flapping movement and torsion synchronised by three Hall sensors. Last year, the more agile and nimble vehicle, ‘BionicSwift’, was designed to make flight manoeuvres as close to reality as possible. Recently, Matloff et al. [9] discovered a clasping structure to explain the cascade mechanism of primary and secondary flight feathers. Based on these observations, Chang et al. [10] developed a semi-biological morphing wing with real flight feathers. During the flight, forty elastically connected flight feathers were controlled using servo-driven wrist and finger joints to achieve a wide range of morphing movements. This aircraft has the highest deformability among all the air vehicles designed so far. However, the wing of this aircraft can only fold in a plane to achieve flight modes such as soaring and turning. Another main research interest is the silent flight of owls, whose wing geometries and properties have evolved over 20 million years to suppress aerodynamic noise [11], [12], [13]. Much work has been devoted to revealing this silent mechanism aimed at the bionic design of airplanes [14]. An aeroelastic design [15] of the airplane wing was inspired based on the observations of the tendency of stresses being distributed as uniformly as possible through its body at any time during wing morphing motions.

Although systematic investigations of ornithopters for bionic bird flight have been undertaken for decades [16], no synthetic aircraft ever designed has achieved a bird's flight abilities. Thus, understanding the extending and twisting motions of bird wings is a prerequisite for designing a bionic flexible wing. Many investigations have contributed to the avian anatomy of the muscles and bones [17]. A broad consensus confirms that bird wing shapes are coordinated under the cooperation of muscles, skeleton, and nerves [18,19]. Although some observations and hypotheses depict anatomic structures, the dynamic changes in wing shape still need to be further investigated based on the coordination mechanism of bones, muscles, and flight feathers. Recent forelimb anatomic studies have shown that different birds may have species-dependent muscles, but the coordination mechanism remains unclear. Musculoskeletal motions were first documented by Bergmann [20]. The wing skeleton was simplified to be a four-bar mechanism ‘drawing parallels’ based on motions of the four prominent bones: humerus, radius, ulna, and carpometacarpus. The parallel arrangement of the ulna and radius can coordinate elbow and wrist joint motions while continuously maintaining its aerodynamic profile, unlike that of non-flying vertebrates. Both radiale and ulnare were neglected in the previous hypothesis but were found in many birds in recent anatomy and myology studies, such as rock pigeons, falcons, and hawks. Amanda [21] measured wing skeletal kinematics of pigeon cadavers using motion tracking marker clusters and micro-computed tomography (mCT) scans. Thereafter, a six-bar mechanism was proposed to fully reconstruct the avian forelimb motions. Even though the wing motions were biologically replicated with the four-bar or six-bar models, the employment of at least four 3-degree-of-freedom (DOF) spherical joints has increased the complexity level, making implementation and control difficult.

Although systematic investigations of ornithopters for bionic bird flight have been carried out for decades, according to our previous research, the morphing abilities of the wings provide birds with extraordinary manoeuvrability, especially raptors. However, little research has been conducted on the morphing abilities of raptor wings. Hence, there is a need for designing flying robots with extraordinary manoeuvrability to better understand the flight of raptors. Therefore, this study aimed to fill this gap in the avian literature and provide a reference for future comparative and functional studies. Finally, a robotic mechanism with morphing abilities was developed based on the skeletons and muscles of a bird of prey.

## 2 Specimen and CT scan experiments

### 2.1 Specimen

**Fig. 1:**
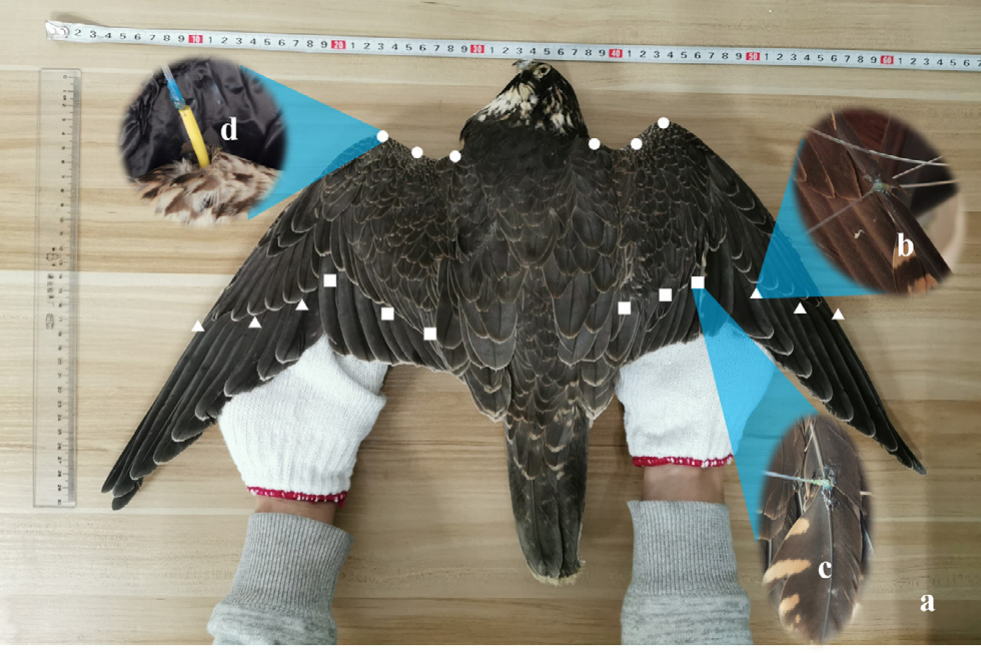
**Carcass of the falcon and fastening operations before scan:** (a) *Falco Peregrinus* with a wingspan of 610 mm; (b–d) operations on the first flight feathers Δ, secondary flight feathers , and cover feather at the leading edge O.

Computed tomography (CT) scan experiments of a *Falco Peregrinus* were conducted because it is one of the world's fastest birds, reaching speeds of 320 km/h before striking its prey. They are strong, fast, and ferocious; they hunt by clenched talons and kill by impact using their high speed and manoeuvrability. However, *Falco Peregrinus* is a second-class nationally protected animal in China, and any sale, purchase, or use of the raptor is forbidden. Hence, live experimentation was impossible due to the threatened and protected nature of the species. Fortunately, a carcass of *Falco Peregrinus*, dead and frozen for a long time, was donated by the Zhejiang Museum of Natural History; significant information about forelimb structure and function was obtained from the study of the specimen. The falcon had no signs of emaciation, decomposition, or other trauma-induced muscle abnormalities. After collection, the carcass was kept at -5 ${\;}^{\circ }$C, warmed and thawed within 3 h, and followed by pre-operations on the specimen, as shown in Fig. 1a.

### 2.2 Suspension system and CT scans

**Fig. 2:**
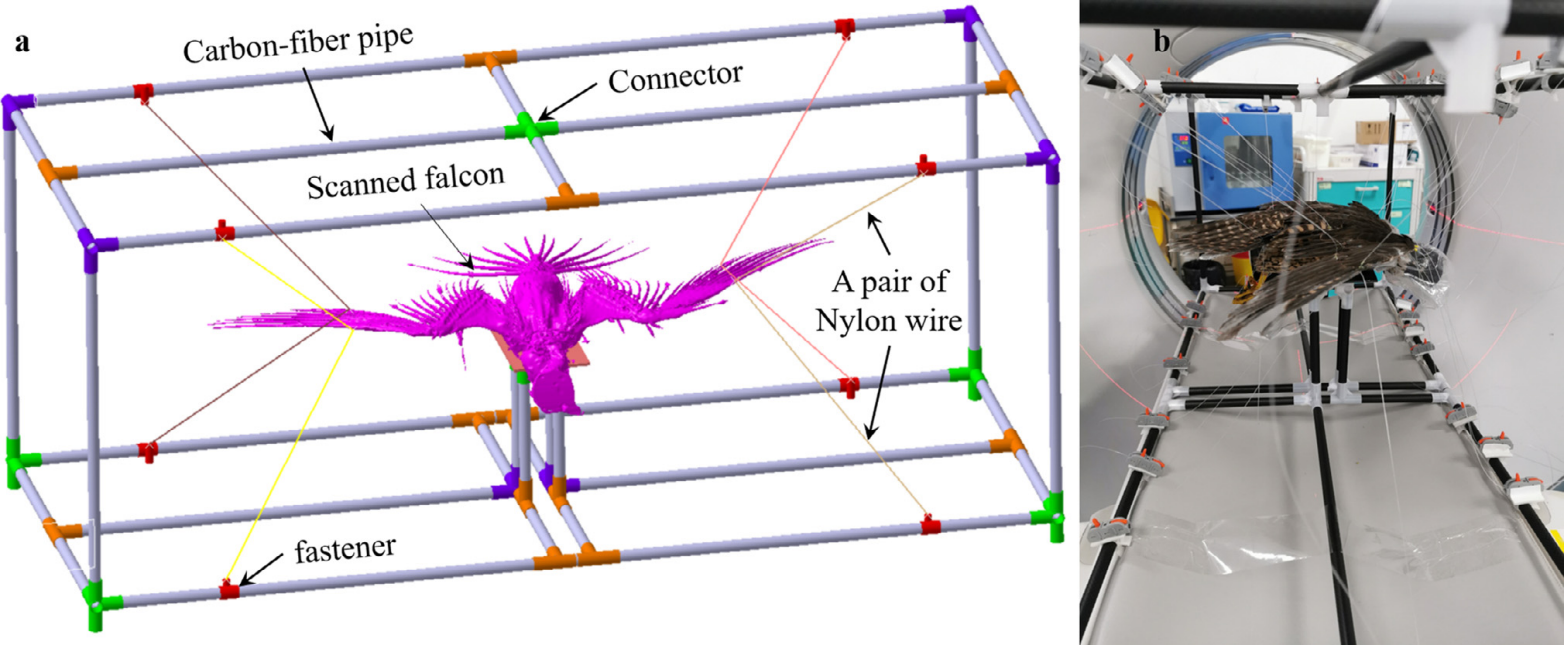
**Rendering of the suspension system for CT scans of the raptor:** (a) Schematic diagram of suspension system; (b) CT scans of *Falco Peregrinus*.

With improved CT resolution, there has been a research proliferation in birds using this technology to visualise the relationship between muscle structures, feathers, and bones [22], [23], [24], [25]. In previous studies, avian carcasses were usually fixed on a bed for scanning. However, they could not change their shape quantitatively. Therefore, fastening the falcon specimen to a flying pose during the CT scans is difficult because (1) the specimen should be fastened and kept undeformed on the moving bed during scanning, (2) various flight attitudes should be included, (3) metal or other similar materials that may have disturbance effects on the X-rays should be avoided, and (4) no direct contact on the specimen is recommended. As shown in Fig. 2a, we designed a suspension system by involving these valuable suggestions by the team of Dr. Cui. In this system, carbon-fibre pipes were connected using T-branch pipes and four-way pipes made of low-density plastic. A testbed was designed to support the carcass at the cube centre, and self-locking nylon cable ties were used to fasten it. Nine pairs of nylon wires were used to suspend each wing to maintain the exact flying pose: three pairs were glued on the leading edge before the humerus and radius; three pairs were glued on the secondary flight feather; three pairs were glued on the flight feather, as shown in Fig. 1b–d.

As a result, a bird suspension system was established. Before the CT scans, the carcass was placed and adjusted to a flight pose, followed by the fastening of all 18 pairs of nylon wires, as shown in Fig. 2b. Each nylon wire was tightened so that each wing could maintain its geometric shape during the entire scanning test. The carcass shapes were manually adjusted to flight postures because the dead *Falco Peregrinus* could not move its wings naturally. Fortunately, the adjusted wing shapes are quite similar to the flight photos under the professional directions of the ornithologists of the Zhongyong Fan team. CT was performed at the affiliated Hangzhou XiXi Hospital (GE Revolution EVO). Thereafter, CT imaging of the entire body was performed. To this end, a clinical scanner was used to obtain a plain CT study at 0.625 mm slice thickness, reconstructed using an auxiliary algorithm. After the scans, the testing bed was cleaned and disinfected to guarantee biosafety.

## 3 Kinematic analysis of the wing

### 3.1 Scans of wing skeleton

**Fig. 3:**
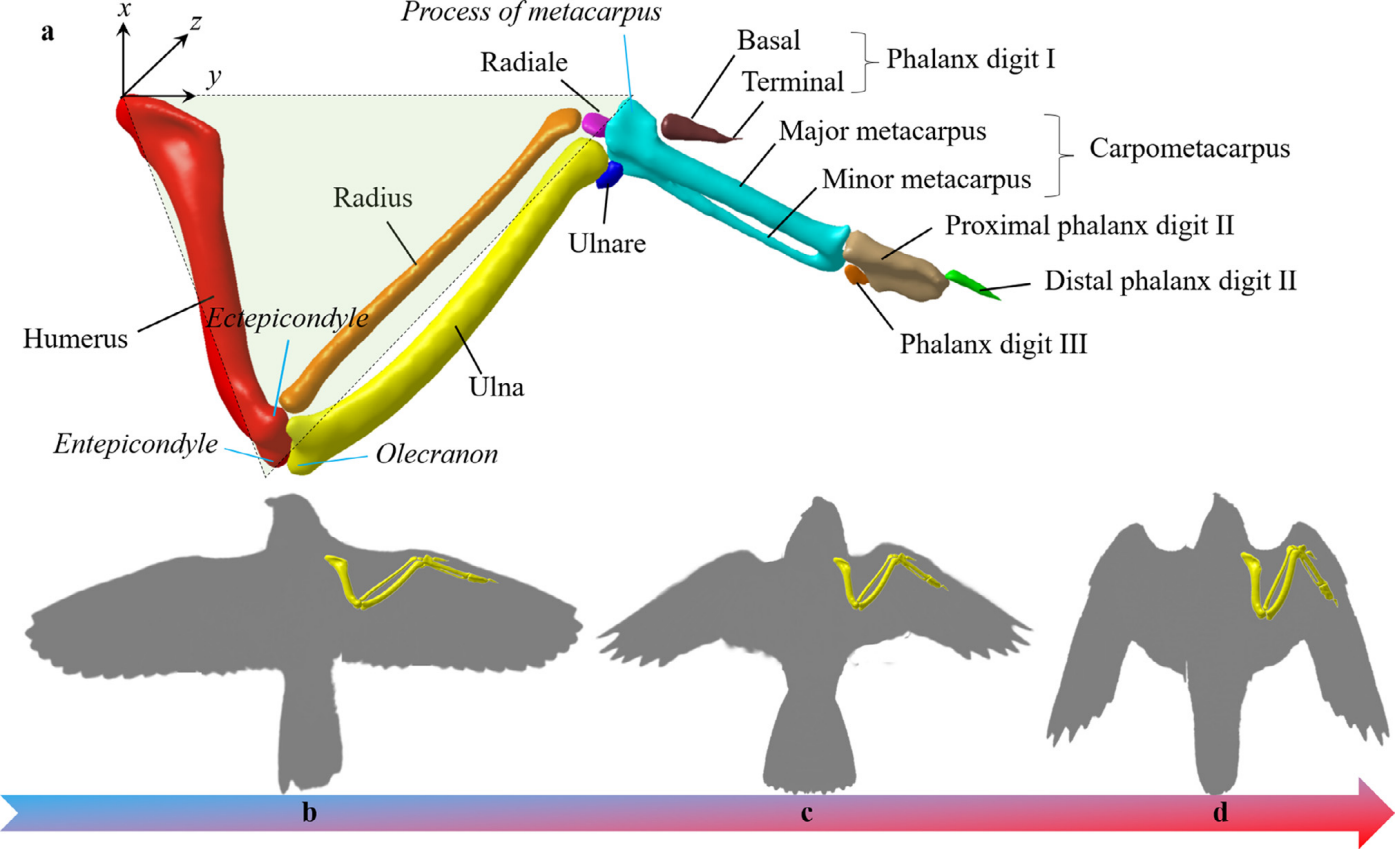
**Forelimb elements in*****Falco Peregrinus*****and three poses of the wings from cruising to diving:** (a) Anatomy of the forelimb elements in *Falco Peregrinus*, and silhouettes in the dorsal aspect of the falcon wing shape with scanned skeleton, (b) extension, (c) half-extension, and (d) extraction.

Raptor wings are sufficiently flexible to change their geometries to maintain efficient aerodynamic performance across a wide range of speeds [26]. Peregrine falcons are hailed as the fastest animal in the world, with recorded horizontal cruising speeds of 65–90 km h^−1^ and reported dive speeds exceeding 320 km h^−1^ [27]. The flight speeds correspond to the status of the wings [28], and the dynamic wing shape changes during flight have emerged as important components of manoeuvrability [29]. Three poses of the wings were studied to study the continuous morphing process from cruising to diving: extension (slow cruising), half-extension, and extraction (fast diving), as shown in Fig. 3b–d.

Motions and CT scans of the skeleton in avian forelimbs with coupled flexion and extension have a long history of investigation [30]. The anatomy and skeletal muscles of the pigeon shoulder joint have been well-characterised, but the anatomical structures of the elbow joint have not been determined until recently [31]. The musculoskeletal elements of the distal wing are usually species-dependent, resulting in a functional ‘block box’; this block box should be precisely investigated for falcons. Understanding the continuous wing shape changes requires observing the relative position of each bone. Thus, a CT scan of the falcon wing was performed to show the relative positions of all ten bones, as shown in Fig. 3a. Like other avian species, the bones of the falcon wing comprise humerus, ulna, radius, carpus, metacarpus, and digits. The skeleton of the wing is characterised by simplifications and reductions in the form of ankylosis, especially at the tip of the limb. Similar to mammals, the humerus forms the skeleton of the brachium, and the ulna and radius form the skeleton of the antebrachium. Conversely, only the ulnar carpal (ulnare) and radial carpal (radiale) bones, originating from the proximal row of the carpal bones, remain in the falcon. In contrast to humans, the metacarpals of the falcon wrist degenerate to major metacarpal and minor metacarpal and are then incorporated into the metacarpus. In addition, digit bones are considerably reduced; digit I (alular digit) and digit III possess only one phalanx, while digit II has two phalanges [32]. As a result, the elbow and carpus function as mutually dependent hinge joints and can therefore be extended or flexed in tandem.

Although comparative analysis of the muscle and skeleton architectures of the forelimb myology has been widely carried out, little quantitative analysis was further investigated or reported. One prerequisite is the lack of coordinates for measuring these relative motions. An intrinsic wing skeleton coordinate was arranged at the root of the humerus of a fully extended wing, as shown in Fig. 3. The *y*-axis lies along the line created by connecting the leading points of the humerus and the metacarpal. A triangular plane was established associated with the olecranon of the ulna, and the *z*-axis was arranged perpendicular to this plane, as shown in Fig. 3a. The humerus, radius, ulna, and carpometacarpus lie primarily in this plane when the wing is fully extended. All the quantitatify analyzes in the following sections are discussed in the proposed coordinate system.

### 3.2 Analysis of wing motion

**Fig. 4:**
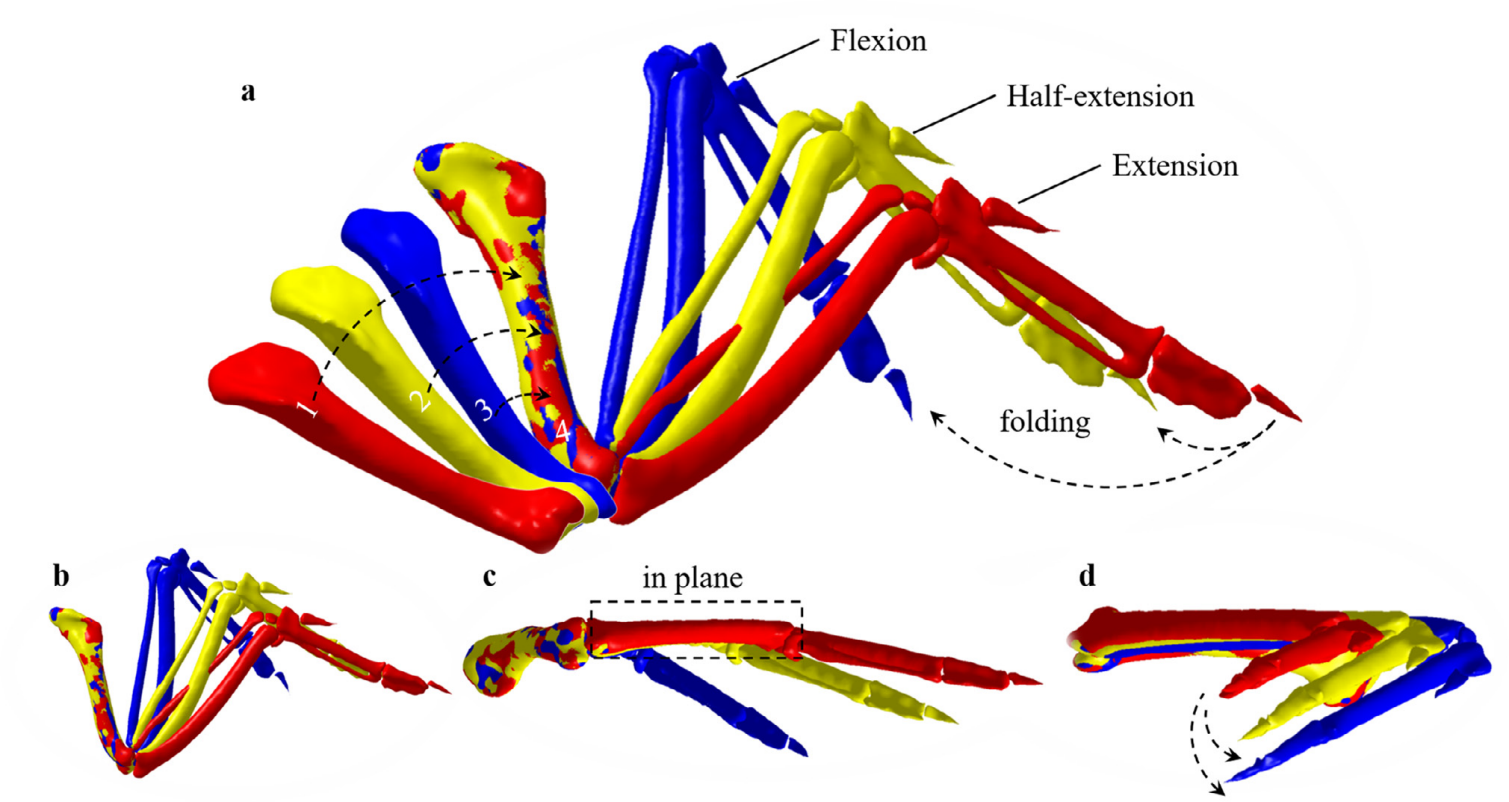
**Point cloud of the scanned wing skeletons:** (a) illustration of three humerus’ (1–3) transformation to the exact position 4 using the proposed optimization method, and the combined skeletons at extension, half-extension, and flexion status; (b–d) dorsal, back, and right view of the three skeletons.

Although only a few studies have depicted and analyzed wing motions [33], measuring the translational and rotational motions in a 2D plane is insufficient to illustrate its 3D motions. Artificial but random operations in modeling are obstacles that still need to be overcome. Therefore, we studied wing motions based on the CT data using a point cloud optimisation approach to determine the centres and axes of rotation for each bone. Both translation variable $T_{k}$ and rotation variable $R_{k}$ were used to represent the relative position of the moving bone *k* in the intrinsic wing skeleton coordinates. All the scanned point data were used to guarantee the fit precision, with 9763 points for humerus and 7323, 3965, 4901, and 1813 points for ulna, radius, metacarpus, and digit II, respectively. The current location of the moving bone after the transformation ($R_{cur},\;T_{cur}$) can be calculated using the following equation:

(1) $$L_{cur}=R_{cur}L_{init}+T_{cur}$$

where $L_{cur}$ and $L_{init}$ are the locations of the moving bones at the current and initial positions, respectively. Thereafter, the average distance between the current and initial status can be obtained as:

(2) $$d=\frac{1}{N}\sum_{j=1}^{N}\min_{1\leq i\leq M}\sqrt{{\left(L_{cur}^{j}-L_{init}^{i}\right)}^{2}}$$

where *M* denotes the point number of the cloud with an extended posture and *N* is the point number of the half-extend or folded cloud. A loop was used to find the minimum value between point $L_{cur}^{j}$ and the point cloud at the initial position. Finally, a global optimisation Eq. 3, using the *MultiStart* method in MATLAB software, was used to find the exact transformation variables ($R_{cur},\;T_{cur}$) that ensured a minimum distance between the two bones. A good agreement is achieved, as shown in Fig. 4a.

(3) $$f\left(R_{cur},\;T_{cur}\right)=min\left(d\right)$$

A falcon can coordinate its wing shape passively using a suite of unique mechanisms; thus, wing skeleton motions of the carcasses represent a total 3D transformation rather than in a 2D plane, as shown in Fig. 4b–d. This is irrefutably proven by the ulna and radius, which form the skeleton of the antebrachium, as in other mammals.

**Fig. 5:**
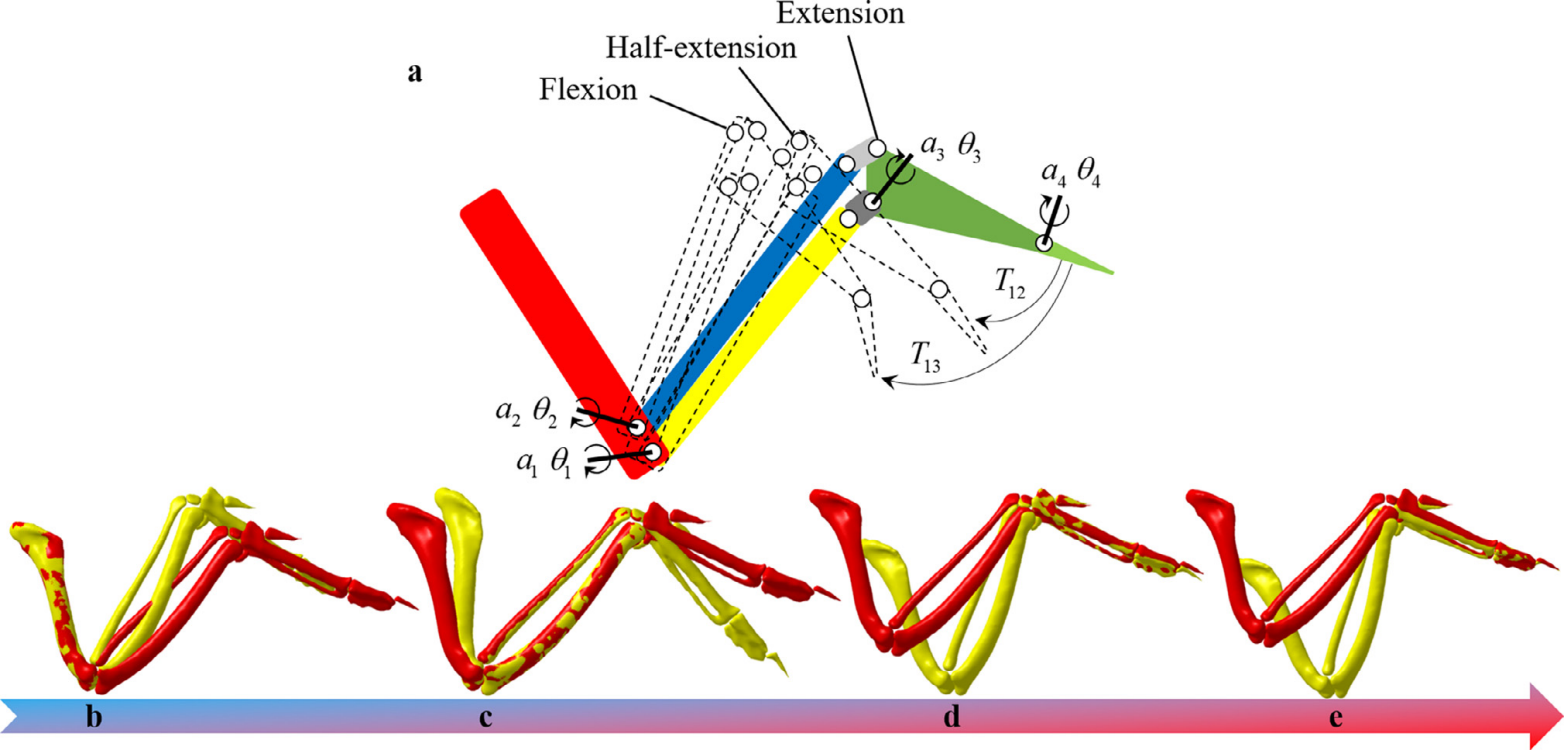
**Skeletal model and the motion measurement procedures of the falcon wing:** (a) Seven-bar skeletal model of the falcon wing; (b–e) the motion measurement procedures using a point cloud optimization approach.

To quantify the full range of the wing motions, a simplified seven-bar skeletal model of the falcon wing, including four main wing bones (humerus, ulna, radius, metacarpus), two wrist bones (ulnare and radiale) [34] and a digit (phalanx digit II), was used to represent the 3D skeletal kinematics, as shown in Fig. 5a, where $a_{i}$ and $\theta_{i}$ represet the rotating axes and angles, respectively, $T_{ij}$ represets the translation from posture *i* to posturee *j*. The distal phalanx digits I and III were also simplified. However, the radiale and ulnare motions were not measured in the current study because these bones are too small to reconstruct at the adopted precision of 0.625 mm. The wing postures were manually adjusted according to the suggestion of Pro. Fan team. According to their empirical experiences on bird flights, each wing was scrupulously adjusted and scanned thereafter. However, these wing postures were unrepeatable; consequently, no repeatability verification was discussed in the current study. The motions (both axis and angular) of the radius and ulna relative to the humerus, carpometacarpus relative to the ulna, and proximal phalanx digit II relative to the carpometacarpus were measured by a step-by-step point cloud fitting approach, as shown in Fig. 5b–e. At the half extension position, a maximum rotation angle of 23.44${}^{\circ }$ was found for the metacarpal; in contrast, only approximately 17${}^{\circ }$ was found for the ulna and radius. A 3D motion of the wing skeleton was proven by the rotating axes that deviated from the *z*-axis, consistent with the 3D folding motions depicted in Fig. 4d. In contrast, a relatively simple motion of proximal digit II was estimated because of its independence from the four main wing bones. Generally, all the rotating angles from extension to extraction are approximately twice the rotation angle from extension to half-extension, as listed in Table 1.

**Table 1:** **Measurements of the wing skeleton during the folding**.

Skeleton		Extension-Half extension (${}^{\circ }$)				Extension-Flexion (${}^{\circ }$)				Axisdeviation
Order	Bone	Axis			Theta	Axis			Theta	

1	Ulna	−0.21	−0.17	0.96	16.42	−0.31	−0.13	0.94	38.83	11.02%
2	Radius	−0.33	−0.31	0.89	17.52	−0.33	−0.19	0.93	38.89	12.09%
3	Metacarpal	0.28	−0.22	0.93	23.44	0.22	−0.11	0.97	55.04	12.66%
4	Proximal digit II	−0.01	0.18	0.98	4.84	−0.12	0.39	0.91	10.02	25.32%

## 4 Bionic design and manufacturing

### 4.1 Optimization of the mechanical joint locations and rotations

**Fig. 6:**
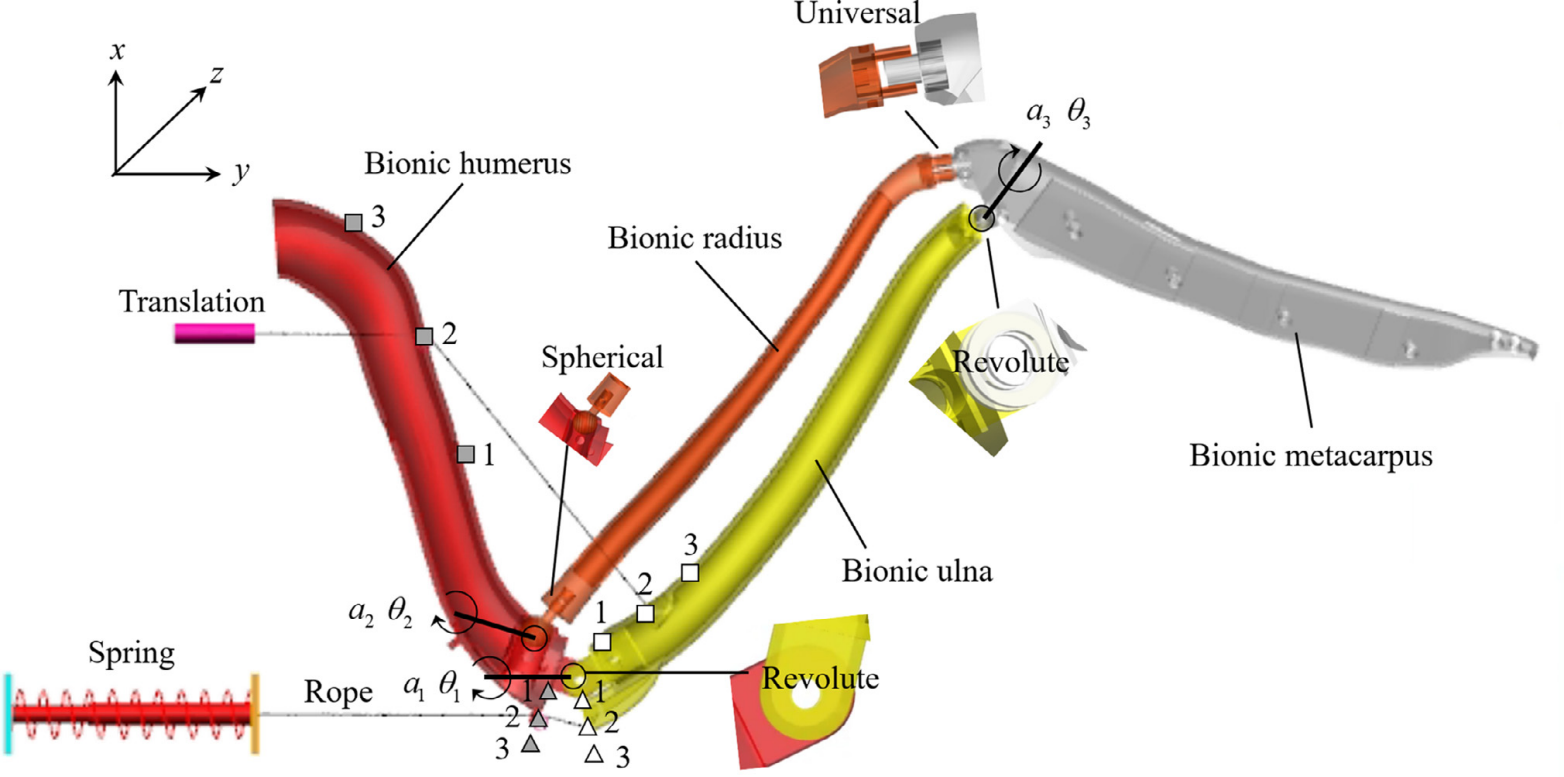
**Bionic wing skeleton with four prominent bones, four joints, ropes, and motor**.

In this section, we attempt to replicate the wing motions using a fabricated mechanism. The multi-bar mechanism paradigm inferred from anatomical studies originated in 1839, and a six-bar model has recently been optimised with high fidelity [20]. However, the previous planar four-bar mechanism did not represent measured skeletal motions owing to its 3D characteristics, whereas the six-bar model was too complex to control for its multiple DOFs and numerous spherical joints [35]. As a compromise, we hypothesised the following possible joint types between the four prominent bones: a revolute joint (1 DOF) connects the distal end of the humerus with the proximal end of the ulna, a spherical joint (3 DOF) connects the distal end of the humerus with the proximal end of the radius, a revolute joint (1 DOF) connects the distal end of the ulna with the proximal end of the metacarpus, and a universal joint (2 DOF) connects the distal end of the radius with the proximal end of metacarpus, as shown in Fig. 6. These joints and bones comprise a bionic wing skeleton with one DOF. The best-fit axes of each joint were also optimised using the global optimisation method *MultiStart* in MATLAB software. The centre locations (C) of each joint, as well as the rotation axis of the two revolute joints (A), were optimised using the following equations:

(4) $$\left\{ \begin{array}{c} f\left(A,C\right)=\min\left(resid\right)\\ resid=\sum_{j=1}^{2}\sum_{i=1}^{3}\sqrt{{\left(\vartheta_{mech}^{ij}-\vartheta_{Falcon}^{ij}\right)}^{2}} \end{array}\right.$$

where subject *i* denotes the *i*th angle listed in Table 1, *j* = 1 denotes the folding procedure from extension to half-extension, and *j* = 2 denotes the procedure from extension to flexion.

**Fig. 7:**
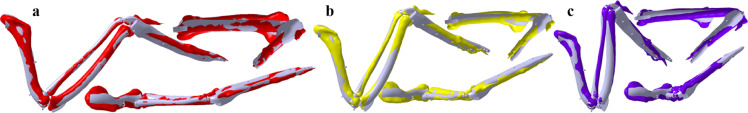
**Comparison of the morphing shapes between the scanned bones of a falcon and the bionic design mechanism:** (a) extension; (b) half-extension; and (c) extraction.

The elbow joint plays a crucial role in wing extending/folding; therefore, revolute joints were used to connect the humerus-ulna and ulna-metacarpus joints. Due to the twisting motions during extension, a spherical joint aligned with a universal joint was used to coordinate these 3D rotations. Comparisons between the bionic design mechanism and falcon bones are shown in Fig. 7. It is shown that a high fidelity was achieved considering that the maximum angle of deviation was less than 1.8${}^{\circ }$ for all joints, as listed in Table 2.

**Table 2:** **Comparison of three main joint rotations between the falcon carcass and bionic mechanical**.

Order	Bionic bone	Extension-Half extension (${}^{\circ }$)			Extension-Flexion (${}^{\circ }$)		
		Falcon	Mechanical	Angle deviation	Falcon	Mechanical	Angle deviation
1	Ulna	16.42	15.54	−0.88	38.83	37.18	−1.65
2	Radius	15.72	17.52	1.80	37.58	38.89	1.31
3	Metacarpal	23.44	22.83	−0.61	55.04	55.65	0.61

**Fig. 8:**
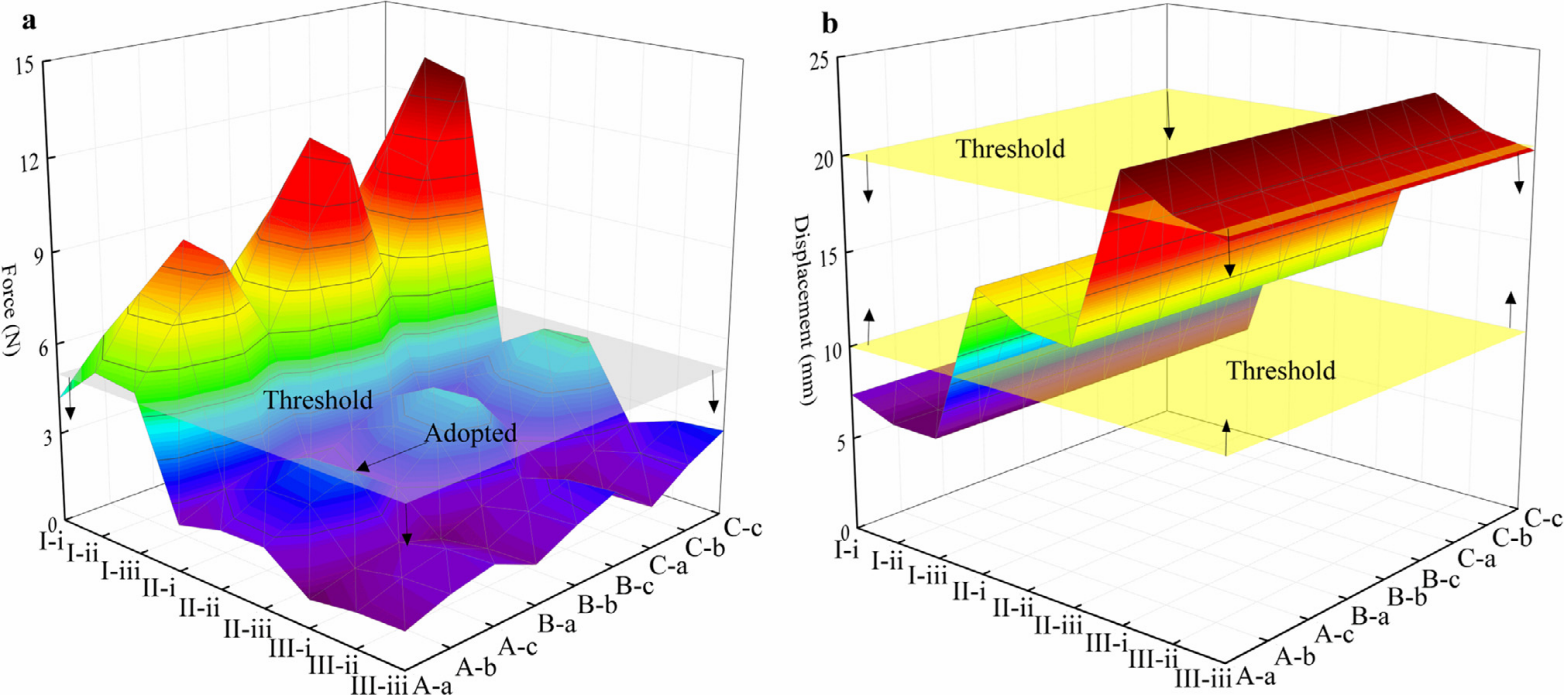
**Comparison of forces and displacements of the bionic wing skeleton during folding:** I-III (), proximal end of the biceps brachii; i-iii (), distal end of the biceps brachii; A-C (), proximal end of the triceps brachii; a-c (), distal end of the triceps brachii; (a) force; and (b) displacement.

Before manufacturing, the forces loaded on the biceps brachii and triceps brachii should be checked carefully to ensure the dynamic performance of both the motor and spring. Thus, three anchor points (represented by  in Fig. 6) were arranged on the humerus to estimate the mechanical performance at the proximal end of the biceps brachii, and three anchor points (represented by ) were arranged on the ulna to study the mechanical performance at the distal end of the biceps brachii. Similarly, three anchor points () and three anchor points () were used to study the mechanical performances of triceps brachii. The quasi-steady multi-body dynamic performances were computed using the ADAMS software. Both the maximum forces and displacements were found when the bionic skeleton was fully folded, as illustrated in Fig. 8. Considering that a large tension or displacement will decrease the dynamic performance of the micro linear actuator, a maximum force threshold of 5 N and displacement threshold ranging from 10–20 mm were used as critical values to select motors and springs. Finally, arrangements II-ii and B-b were adopted in the current research.

### 4.2 Bionic manufacturing and experimental tests

**Fig. 9:**
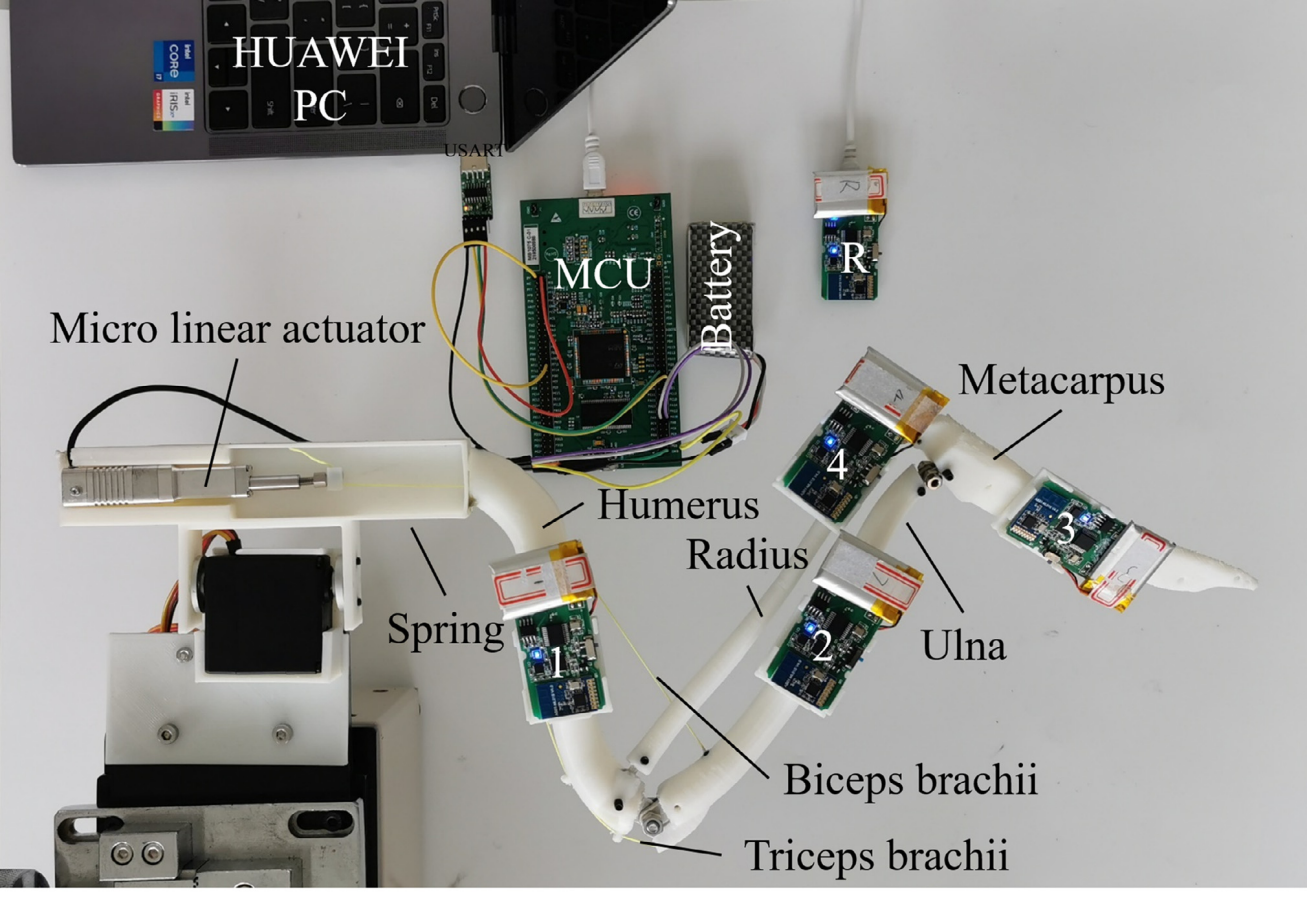
**Fabricated wing skeleton and the motion capture system to measure folding motions**.

A simpler 3D four-bar mechanism with four revolute joints cannot move along the bone motion paths, and a four-bar mechanism with four spherical joints is recommended to track the measured skeletal kinematics. A compromise simulated result was achieved, though less accurate than that of the six-bar model [20]. However, 6 DOF require at least 12 wires to sustain their morphing shape, which is too complex to control. To achieve easy but stable control, a mechanism with the proposed spherical universal revolute joints was manufactured to verify the bionic design, as shown in Fig. 9.

The four prominent bionic bones were printed on a 3D printer (0.05 mm precision, L8-410, 10–150 mm/S, Leader 3D, China). The size of the printer's build plate was 410 mm × 410 mm × 500 mm. All bones were printed with a 1.75 mm layer thickness out of polylactic acid material (PLA plus, density 1.23 g/cm^3^, and tensile strength 63–74 MPa), resistant to common disinfectants and environmentally stable [36], [37], [38]. A micro linear actuator container and IMU supporters were printed to support the bones for testing. All the printed parts were adjusted and connected using spherical, universal, and revolute joints; they were designed and manufactured with steel. The critical length between the neighbouring joints was measured to eliminate misalignment during adjustment. Protective earthing (PE) wires were used to mimic both biceps brachii and triceps brachii, and they established a stable connection between the humerus and ulna to form a pair of antagonisms. During tests, the micro linear actuator (LA16-021D/P, maximum pull 70 N, precision 0.03 mm, Inspire-robots, China) drove the bones to move through the PE wires. An MCU (STM32F429) was used to control the wing skeleton motion in an open-loop manner that modeled the coupled motion between the elbow and wrist. The wing in its extended position was referenced to measure the rotational angles during folding based on the motion capture data (MPU9250).

**Fig. 10:**
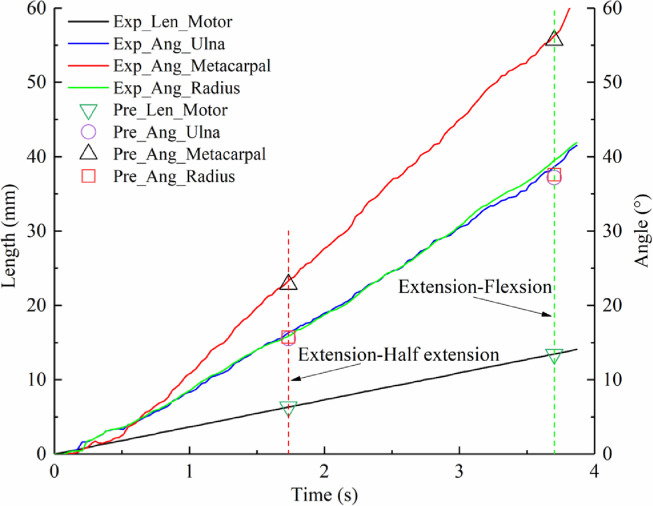
**Measured rotational angles of the three prominent bones and a comparison to the predicted data**.

The rotating angles of each bone were considered as critical values; thus, the measured angles of the fabricated wing skeleton (prefix, Exp_Ang) are illustrated in Fig. 10. A linear control strategy of 4 mm/s was used to drive the micro linear actuator (Exp_Len_Motor). It is shown that the rotating angles of the fabricated metacarpal are consistent with the predicted values (prefix, Pre_Ang) in the mechanical design discussed in Section 4.1, with a maximum deviation of 0.59${}^{\circ }$, whereas a larger angle deviation was found at 1.91${}^{\circ }$ for the ulna and radius. Angle deviations of the simplified 4-bar model indicate that radiale and ulnare may be necessary to model wing morphing accurately. Above all, the simplified 4-bar model achieved a high resolution to replicate the wing folding motions by directly optimising their joint locations and types. Combined with its simplicity and robustness, we estimate this is an enlightening bionic inspiration for morphing aircraft design.

## 5 Conclusion

The functional properties of muscles and their intrinsic mechanics and kinematics of flight have been widely investigated. However, it was accomplished without consulting a scanned database for raptors because such a database was either quite incomplete to use or even non-existent. Particularly, the wing skeleton database of *Falco Peregrinus* has not been examined yet. Therefore, to provide a reference for future comparative and functional studies, the forelimb of a bird of the prey *Falcon peregrinus* was studied by imaging the wings with different postures. The wing skeleton of the bird was consecutively CT-scanned from folded posture to fully extended posture, and the behavior and contribution of the four prominent bones during the process of folding to unfolding were determined. Finally, a bionic wing mechanism with a simple four-bar linkage is designed and fabricated to implement folding/unfolding motions with high fidelity. In this study, we evaluated the following:

(1) A carbon-fibre suspension system with nylon wires (nonmetal material) was utilized to fasten and maintain the hanging specimen to mimic the motion of a bird wing. The suspension system is quite suitable for bird morphing posture studies, such as CT scans, 3D shape-scans, motion capture, etc.

(2) The myology of the forelimb skeleton was studied in the current research to analyze the kinematics of the wing quantitatively. An intrinsic coordinate system at the base of the humerus was defined, and the 3D motions of the forelimb bones were represented by translations and rotations under the coordinates. The falcon-wing skeleton was examined in detail, and three critical postures were scanned during folding to obtain the CT-scanned point clouds. The centres and axes of rotation of each bone were determined to mimic wing motion quantitatively using a pose optimisation method. Thereafter, the kinematics of the wings were quantitatively analyzed.

(3) The ten forelimb bones were simplified to an artificial mechanism as a seven-bar model to represent 3D skeletal kinematics. Using a step-by-step point-cloud fitting approach, the relative axial and angular motions of the four prominent bones were extracted, followed by quantitative measurements of the wing skeleton during the folding/unfolding motions. Inspired by these observations, a bionic mechanism with revolute, universal, and spherical joints was proposed, and the locations and rotations of each joint were optimised using a global optimisation method to resemble the actual motions of the forelimb.

(4) Finally, a 3D four-bar mechanism (robotic-like wing) is fabricated using a 3D printer. As a good outcome, a rotational comparison of the three main joints between the falcon carcass and the artificial mechanism was provided. The mechanical performance of the artificial mechanism during folding/unfolding motions, such as maximum forces and maximum displacements, was computed and optimised to guarantee structural integrity. In addition, the rotational angles of the three prominent bones were measured and compared with the predicted values to show that the proposed simple four-bar model can replicate folding/extending motions with high fidelity.

The observations of the current study can be a useful guide for the morphological analysis of birds and an enlightening inspiration for the bionic design of morphing aircraft in the future.

## Declaration of competing interest

The authors declare that they have no conflicts of interest in this work.

## Appendix Supplementary materials

Image, application 1
