# Supplementary material for: Quantitative analysis of the morphing wing mechanism of raptors: Analysis methods, folding motions, and bionic design of Falco Peregrinus
Source: Fundam Res. 2022 Apr 29;4(2):344–52. doi: 10.1016/j.fmre.2022.03.023 (PMC11630695; doi:10.1016/j.fmre.2022.03.023)
Supplement: Supplementary file 1 [file mmc1.pdf]

Quantitative analysis of the morphing wing mechanism of ~~birds of prey~~raptors: analysis methods, folding motions, and bionic design of *Falco peregrinus*

Abstract:

Raptors can change the shape and area of their wings to an exceptional degree in a fast and efficient ~~way~~manner, surpassing other birds, insects, or bats. Some researchers have focused on the functional properties of ~~the~~ muscle~~-~~ skeletons, ~~the~~ mechanics, and ~~the~~ flapping robot design. However, the wing motion of ~~bird~~the birds of prey has not been measured quantitatively, and ~~man-made~~synthetic bionic wings with morphing abilities ~~just like a raptor is~~similar to raptors are far ~~away~~ from reality. ~~Thus~~Therefore, in the current study, a 3D suspension system for holding bird carcasses was designed and fabricated ~~in order~~ to fasten the wings of *~~F~~falco~~n~~ peregrinus* with a series of morphing postures~~ in the current researches. Then~~. Subsequently, the wing skeleton of the falcon was scanned during extending motions using ~~CT (~~the computed tomography~~)~~ (CT) approach to obtain three consecutive poses. ~~Thereafter~~Subsequently, the skeleton was reconstructed to ~~show how~~identify the contribution of the forelimb bones ~~contributed~~ to the extending/folding motions. Inspired ~~from~~by these findings, we propose~~d~~ a simple mechanical model with four bones ~~that can~~ to form ~~the~~a wing~~-~~morphing mechanism using ~~a~~the proposed pose optimi~~z~~sation method. Finally, a bionic wing mechanism was implemented to imitate the motion of the falcon wing~~, which was~~ divided into inner and outer wings with folding and twisting motions. The ~~result shows~~results show that the proposed four-bar mechanism can track ~~the three-dimensional~~high-fidelity 3D bone motion paths~~ with a high fidelity~~.

~~Key words~~Keywords: *Falco peregrinus*; raptor; suspension system; CT scan; ~~wing motion;~~ bionic wings; four-bar mechanism.

1 Introduction

~~The flying~~Flying birds in nature can use flapping wings to ~~move through the~~gracefully and dexterously traverse terrestrial, aerial, and aquatic environments~~ gracefully and dexterously ~~ [1]. As concluded in our previous research~~es~~, the key to ~~this ~~high manoeuvrability in bird flight lies not in the static aerodynamic performances~~,~~ but ~~the way ~~in ~~which~~how the wing morphs to change the flying situations [2,~~-~~3]. However, no aircraft has yet been designed ~~achieves the~~to achieve morphing abilities~~ like~~ of ~~, such as ~~birds. Therefore~~us~~, more attention should be paid ~~back ~~to the myology and anatomy of bird wings, as long as ~~its~~they have a bionic design.

Wing morphing, ~~as ~~the key to manoeuvrability flight, broadens the excellent performance of birds,~~,~~ ~~enabling~~ ~~and ~~enablinges them to fly faster and adjust their attitude more effectively [4–~~-~~6]. Therefore, the flying robot design inspired ~~from~~by flying birds has become ~~one of the hottest topics~~a popular topic in mechanical engineering. An Eagle simulator model, with a weight of ~~425g~~425 g and a wingspan of ~~107cm~~107 cm, was designed to study and verify relevant aerodynamic parameters during flight [7]~~,~~. Gerdes et al. [8] fabricated the pioneering flight, ~~"~~'Robo Raven~~"~~', which had a ~~wing span~~wingspan of ~~150cm~~150 cm and a weight of ~~690g. The~~690 g. A bird-inspired platform was studied to verify that independent wing control ~~endowed the potential to~~can provide a greater flight envelop~~ [8]~~. A ~~state -of -the art~~modern flapping wing air vehicle, ~~"Smart Bird",~~'SmartBird', was developed by the Festo ~~Company, it~~company. It is driven by two servo motors, ~~and~~with the flapping movement and torsion ~~can be ~~synchroni~~z~~sed by three Hall sensors. Last year, the more agile and nimble vehicle, ~~"BionicSwift" was also released, it is~~'BionicSwift', was –designed to ~~execute the~~make flight manoeuvres as ~~true to life~~close to reality as possible~~. was also released~~. Recently, ~~Laura Y. ~~Matloff~~,~~ et al. [9] discovered ~~the clasp~~a clasping structure to explain the cascade mechanism of primary and secondary flight feathers~~ [9]~~~~,~~. Based on these observations, ~~Eric ~~Chang [10] developed a semi-biological morphing wing with real flight feathers. During the flight, ~~40 ~~forty elastically connected flight feathers were controlled ~~through~~using servo-driven wrist and finger joints to achieve a wide range of morphing movements. This~~ is the~~ aircraft ~~with~~has the highest deformability ~~that was~~~~has~~among all the air vehicles ~~been ~~designed so far. However, the wing of this aircraft can only fold in a plane to achieve flight modes such as soaring and turning. Another main research interest is the silent flight of owls, whose wing geometries and properties have ~~been ~~evolved over 20 million years to suppress ~~the ~~aerodynamic noise [11–~~-~~13]. Much work ~~was contributed~~has been devoted to revealing this silent mechanism aimed at the bionic design of airplanes [14]. ~~Based~~An aeroelastic design [15] of the airplane wing was inspired ~~[15] ~~based on the observations of the tendency ~~that the~~of stresses ~~are ~~being distributed as uniformly as possible through its body at any time during wing morphing motions~~, an aeroelastic design of the airplane wing was inspired [15].~~.

Although systematic investigations ~~on~~of ornithopters ~~to~~for bionic bird flight have been ~~carried out~~undertaken for decades [16], ~~however, ~~no ~~man-made~~synthetic aircraft~~s~~ ever designed has achieved ~~the~~ a bird's flight abilities. Thus, understanding ~~how ~~the extensions and twists ~~the~~aof bird wings ~~extends and twists ~~is ~~the~~a prerequisite ~~to design~~for designing a bionic flexible wing. Many investigations have ~~been~~ contributed to the avian anatomy ~~structures ~~of the muscles and bones [17]. A broad consensus ~~has been reachieved ~~confirms that ~~the ~~bird wing shapes are coordinated under the co~~rp~~operation~~s~~ of muscles, skeleton, and nerves [18,~~-~~19]. ~~T~~Although some observations and hypothes~~ie~~es depict ~~the ~~anatomic structures, ~~a deep understanding of ~~the dynamic changes ~~of the~~in wing shape still need~~s~~ to be further investigated based on the coordination mechanism of ~~the ~~bones, muscles, and flight feathers. Recent forelimb anatomic studies ~~show~~have shown that different birds may have ~~a specie~~species-dependent muscles, but ~~its~~the coordination mechanism ~~is still~~remains unclear. Musculoskeletal motions were first documented by Bergmann~~,~~.~~;~~ T~~t~~he wing skeleton was simplified to be a four-bar mechanism 'drawing parallels' based on

motions of the four mainprominent bones: humerus, radius, ulna, and carpometacarpus. The parallel arrangement of the ulna and radius can coordinate elbow and wrist joint motions while continuously maintaining its aerodynamic profile [20], which is different withfromunlike that of non-flying vertebrates. Both radiale and ulnare were neglected in the previous hypothesis but were found in many birds in recent anatomy and myology researchesstudies, such as rock pigeons, falcons, and hawks, et al. And. -Amanda K.Stowers [21] measured wing skeletal kinematics of pigeon cadavers using motion tracking marker clusters and micro-computed tomography (mCT) scans. Thereafter, a six-bar mechanism was proposed to fully reconstruct the avian forelimb's motions of the avian forelimb.. Even though the wing motions were biologically replicated with the four-bar model or six-bar model biologically, models, the employment of at least four 3-degree-of-freedom (DOF) spherical joints has increased the complexity level, which makes it difficult to implementmaking implementation and control difficult.

Although systematic investigations onof ornithopters tofor bionic bird flight have been carried out for decades, it isaccording to our previous research, the morphing abilities of wing that enablesthe wings enableprovide a birds with extraordinary manoeuverability, especially for birds of prey, according to our previous researches..raptors. However, little research has been contributed to conducted on the morphing abilityies of raptor's wingraptor wings. To getobtain aHencen, there is a need for designing outstanding design offor flying robots with an extraordinary manoeuverability to , we may better understand the flight of raptors. The aim ofTherefore, this study was, therefore,aimed to fill this gap in the avian literature and to provide a reference for future comparative and functional studies. Finally, a robotic mechanism with morphing abilities was inspireddeveloped based on the skeletons and muscles of a bird of prey.

2 Specimen and CT scan experiments

2.1 Specimen

Computed tomography (CT-) scan experiments of a *Falco peregrinus* were studied forconducted because it is one of the world's fastest birds, which can reaching speeds - a tremendous speeds of 320 Kmkm/h before striking theirits prey. They are also strong, fast, but and ferocious,; they hunt by clenched talons and kill by impact with resort tousing itstheir high speed and manoeuverability. However, *Falco Peregrinus* is one of thea second-class nationally protected animals ofanimal in China, and any sale, purchase, or use of the raptor is forbidden. DueHence, live experimentation was impossible due to the threatened and protected nature of the specie, live experimentation could not be done.species. Fortunately, a carcass of Falco peregrinus, dieaed and frozen for a long time, was donated by the Zhejiang Museum of Natural hHistory;, and a great deal ofsignificant information about forelimb structure and function can bewas obtained from outthe study of the specimen. The falcon was examined to havehad no signs of emaciation, decomposition, or other trauma-induced muscle abnormalities. After collection, the carcass was kept at -5°C, then warmed, and thawed within 3 hoursh, and followed withby pre-operations on the specimen, as shown in Fig. 1 (a).

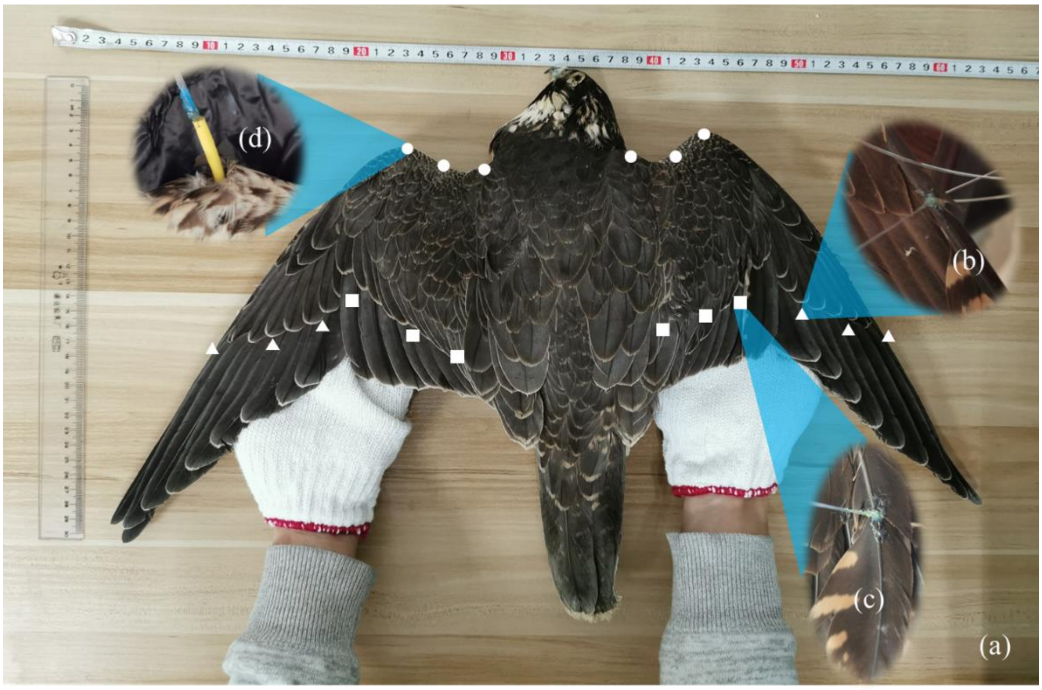

Fig. 1 Carcass of the falcon and fastening operations before scan: (a) *Falco Peregrinus* with a ~~wing span~~wingspan of ~~610mm~~610 mm; (b~~-~~–d) operations on the first flight feathers △, secondary flight feathers □, and cover feather at the leading edge ○.

2.2 Suspension system and CT scans

~~As the~~With improved CT resolution~~ of computed tomography (CT) has improved~~, there has been a research proliferation ~~of research~~in birds using this technology ~~in birds~~ to visuali~~z~~se the relationship ~~among~~between muscle structures, feathers, and bones [22~~-~~–25]. In previous ~~researches~~studies, avian ~~carcass was~~carcasses were usually fixed on a bed for scanning~~ but unable to~~. However, they could not change ~~its~~their shape quantitatively. Therefore, ~~how to~~ fastening the falcon specimen to a flying pose during the CT scans ~~puzzled us,~~ is difficult because~~ that:~~ (1) the specimen should be fastened and kept undeformed on the moving bed during scanning,~~;~~ (2) various flight attitudes should be included,~~;~~ (3) metal~~,~~ or other similar material~~,~~s that may have disturbance effects on the X-rays~~,~~ should be avoided,~~;~~ and (4) no direct~~-~~ contact on the specimen ~~was~~is recommended. ~~Involved those~~As shown in Fig. 2 (a), we designed a suspension system by involving these valuable suggestions by the team of ~~Doctor~~Dr. Cui~~, we designed a suspension system as shown in Fig. 2 (a).~~. In th~~e~~is system, carbon-fib~~er~~re pipes were connected using T-branch pipes and four-way pipes made ~~with~~of low~~-~~-density plastic. ~~At the cube center, a~~A testbed was designed to support the carcass ~~body~~at the cube centre, and self-locking nylon cable ties were used to fasten ~~the body. In order to~~it.~~ To maintain the exact flying pose,~~ ~~9~~N~~n~~ine pairs of nylon wires were used to suspend each wing to maintain the exact flying pose:~~,~~ ~~where 3~~three pairs were glued on the leading edge before the humerus and radius;~~,~~ ~~3~~three pairs were glued on the secondary flight feather;~~,~~ ~~3and~~ three pairs were glued on the flight feather, as shown in Fig. 1 (b~~-~~–d).

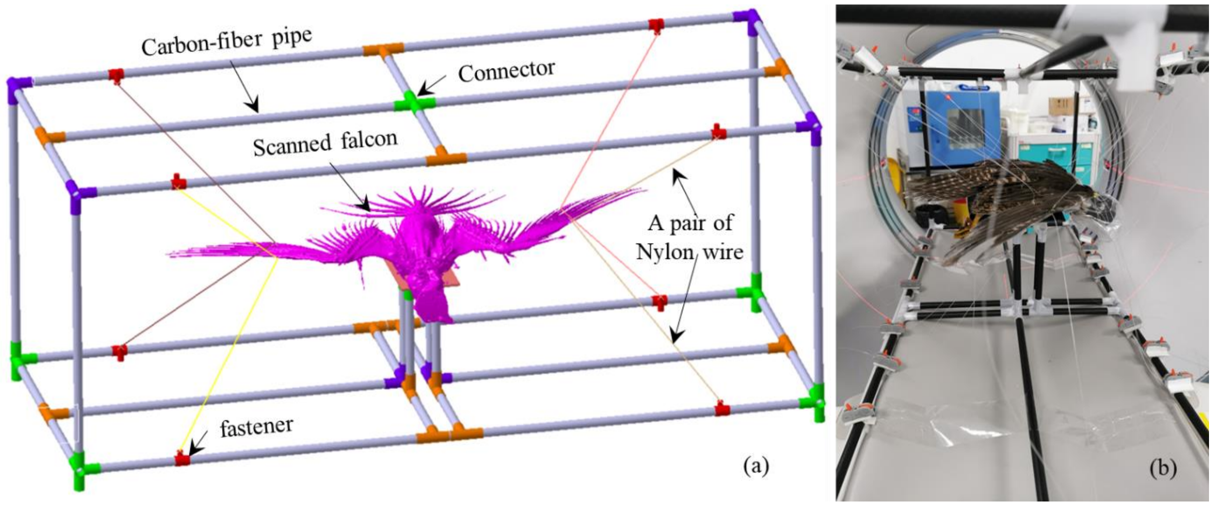

Fig. 2 Rendering of the suspension system for CT scans of the ~~bird of prey~~raptor.

As a result, ~~the~~a bird suspension system was established. Before the CT scans, the carcass was placed and adjusted to a flight pose, followed ~~with~~by the fastening of all ~~the~~ 18 pairs of nylon wires, as shown in Fig. 2 (b). Each nylon wire was tightened so that each wing ~~can~~could maintain its geometr~~y~~ic shape ~~undeformed~~ during the ~~whole~~entire scanning test~~s~~. ~~It should be noted that~~ T~~t~~he carcass shapes were manually adjusted to particular flight postures ~~manually,~~ because ~~that~~ the dead *Falco Peregrinus* ~~cannot~~could not move its wings naturally. Fortunately, the adjusted wing shapes are quite similar to the flight photos under the professional directions of the ornithologists of the Zhongyong Fan team. ~~The~~ CT ~~scans were~~was performed at the affiliated Hangzhou XiXi Hospital (GE Revolution EVO). Thereafter, ~~computed tomography~~CT imaging of the entire body was performed. To this end, a clinical scanner was used to obtain a plain CT study at 0.625 mm slice thickness, ~~which was~~ reconstructed using an auxiliary algorithm. After the scans, the testing bed was cleaned and disinfected to guarantee ~~the bio-safety~~biosafety.

3 Kinematic analysis of the wing

3.1 Scans of Wing skeleton

Raptor wings are sufficiently flexible ~~enough~~ to change their geometries to maintain efficient aerodynamic performance~~s~~ across a wide range of speeds [26]. ~~The peregrine~~Peregrine falcons ~~is~~ are hailed as the fastest animal in the world, with recorded horizontal cruising speeds of 65~~-~~–90 km h^{-1} and ~~with~~ reported dive speeds ~~in excess of~~exceeding 320 km h^{-1} [27]. The flight speeds correspond to the status of the wings [28], and the dynamic wing shape ~~changing in~~changes during flight ha~~s~~ve emerged as ~~an~~ important ~~component in maneuverability~~components of manoeuvrability [29]. Three poses of the wings were studied ~~In order to~~ ~~To~~to study the continuous morphing process from cruising to diving~~, three poses of the wings were studied which were~~: extension (slow cruising), half-extension, and extraction (fast diving), as shown in Fig. 3 (b~~-~~–d).

Motions and CT scans of the skeleton in avian forelimbs with coupled flexion and extension have a long history of investigation [30]. The anatomy and ~~muscleskeleton~~skeletal muscles of the pigeon shoulder joint have been well-characteri~~z~~sed, but the anatomical structures of the elbow joint ~~were~~have not ~~figured out~~been determined until recentl~~y~~ly [31]. ~~It is found that T~~Tthe musculoskeletal elements of the distal wing are usually species~~-~~-dependent, resulting in a functional ‘block box’; ~~which~~ this block box should be precisely investigated ~~precisely~~ for ~~the~~ falcons. Understanding the continuous wing shape changes requires ~~an observation of~~ observing the relative position of each bone. Thus, a CT scan ~~study~~ of the falcon wing was ~~made~~performed to show the relative positions of all ~~the 10~~ten bones, as shown in Fig. 3 (a). Like other avian species, the bones of the falcon wing ~~are comprised of~~comprise humerus, ulna, radius, carpus, metacarpus, and digits. The skeleton of the wing ~~was characterized~~is characterised by simplifications and reductions in ~~a~~the form of ankylos~~e~~is, especially at the tip of the limb. ~~As in~~Similar to mammals, the humerus forms the skeleton of the brachium, and the ulna and radius form the skeleton of the antebrachium. ~~On the other hand~~Conversely, only the ulnar carpal ~~bone~~ (ulnare) and ~~the~~ radial carpal ~~bone~~ (radiale) ~~remain in the falcon, which originate~~bones, originating from the proximal row of the carpal bones~~. On~~, remain in the ~~contrary~~falcon. In contrast to humans, the metacarpals of the falcon wrist degenerate to major metacarpal and minor metacarpal~~,~~ and are then ~~are~~ incorporated into the metacarpus. ~~Also~~In addition, digit bones are considerably reduced~~, the~~; digit I (alular digit) and digit III possess only one phalanx ~~respectively~~, while ~~the~~ digit II has two phalanges [32]. As a result, the elbow and carpus function as mutually dependent hinge joints~~,~~ and can therefore ~~can~~ be extended or flexed in tandem.

~~It should be noted that, though~~Although comparative analysis of the muscle and skeleton architectures of the forelimb myology ha~~ve~~s been widely carried out, ~~however,~~ little quantitative analysis was further investigated or reported. One ~~of the prerequisites~~prerequisite is the lack of ~~coordinate to measure~~coordinates for measuring these relative motions. ~~Here, an~~An intrinsic wing skeleton coordinate was arranged at the root of the humerus of a fully extended wing, as shown in Fig. 3. ~~Where the~~The y~~-~~-axis lies along the line ~~which was~~ created by connecting the leading points of the humerus and the metacarpal. ~~Associate with the olecranon of ulna, a~~A triangular plane was established~~,~~– associated with the olecranon of the ulna, and the z~~-~~-axis was arranged perpendicular to th~~e~~is plane, as ~~denoted~~shown in Fig. 3 (a). ~~It is shown that the~~The humerus, radius, ulna, and carpometacarpus lie primarily in this plane when the wing is fully extended. All the quantitative analys~~i~~es in the following sections ~~is~~are discussed in the proposed coordinate system.

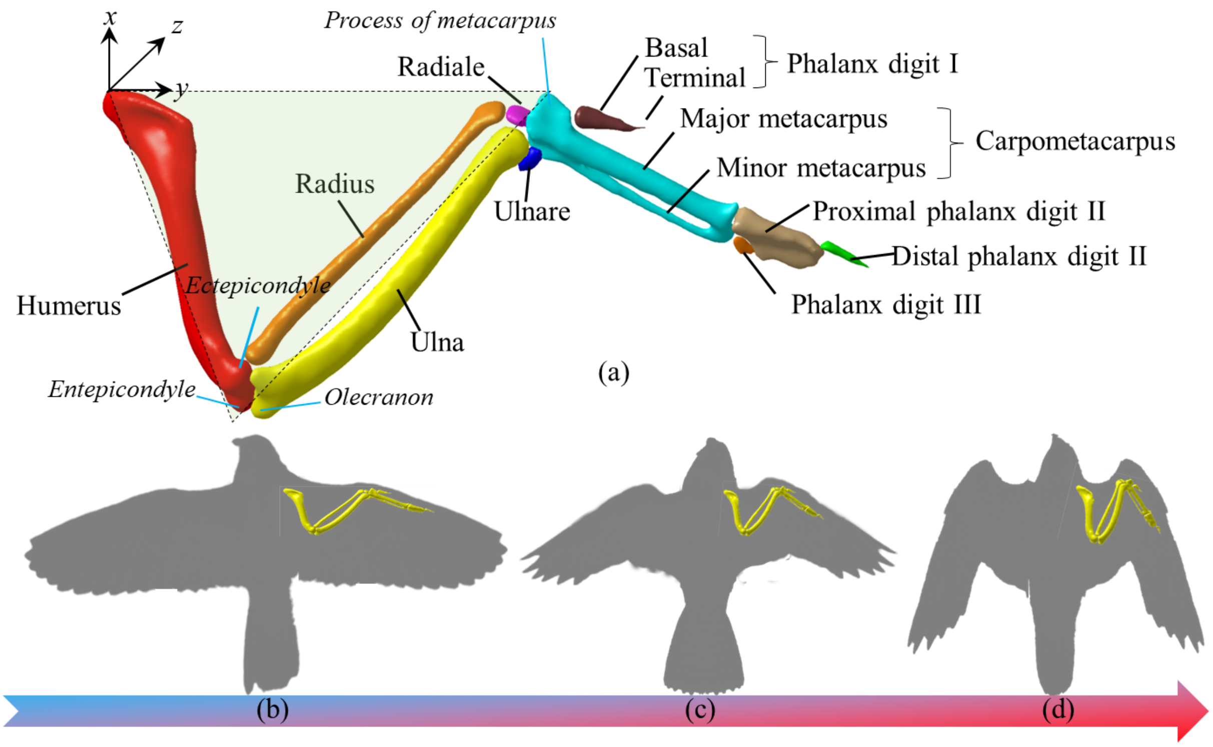

Fig. 3 Anatomy of the forelimb elements in *Falco Peregrinus* (a), and silhouettes in the dorsal aspect of the falcon wing shape with scanned skeleton: (b) extension, (c) half-extension, and (d) extraction.

3.2 Analysis of wing motion

~~Though the current~~ Although only a few ~~researcheses~~studies have depicted and analy~~z~~sed wing motions [33], ~~however,~~ measuring the translational ~~motion~~ and rotational motions in a 2D plane is insufficient to illustrate its 3D motions~~, and artificial~~. Artificial but random~~ness~~ operations in modelling are ~~still the~~ obstacles that still need to be overcome. Therefore, we studied wing motions based on the CT data using a point cloud optimi~~z~~sation approach to determine the cent~~e~~res and axes of rotation for each bone. Both translation variable T_k and rotation variable R_k were used to represent the relative position of the moving bone k in the intrinsic wing skeleton coordinates. ~~To guarantee the fit precision,~~ ~~A~~All the scanned point data were used to guarantee the fit precision, ~~where~~with 9,763 points for humerus and 7323, 3965, 4901, and 1813 points for ulna, radius, metacarpus, and digit II, respectively. The current location of the moving bone after ~~a~~the transformation (R_{cur}, T_{cur}) can be calculated using the following equation:

$$L_{cur} = R_{cur} L_{init} + \sout{T_{cur}} T_{cur}.$$

~~W~~where L_{cur} and L_{init} are the locations of the moving bones at the current ~~position~~ and ~~the~~ initial positions, respectively. Thereafter, ~~an averaged~~the average distance between the current and ~~the~~ initial status can be obtained as

$$d = \frac{1}{N} \sout{\sum_{j=1}^{N} \min_{1 \le i \le M} \sqrt{(L_{cur}^{j} - L_{init}^{i})^2}} \sum_{j=1}^{N} \min_{1 \le i \le M} \sqrt{(L_{cur}^{j} - L_{init}^{i})^2}\ , \qquad (1)$$

~~W~~where M denotes the point number of the cloud with an extended posture~~,~~ and N is the point number of the half-extend or folded cloud. ~~Obviously, a~~A loop was used to find the minimum value between ~~the~~ point L_{cur}^{j} and the point cloud at the initial position. Finally, a global optimi~~z~~sation equation (2), ~~with~~using the *MultiStart* method in MATLAB software, was used to find the exact transformation variables (R_{cur}, T_{cur})

that ensured a minimum distance ~~of~~between the two bones. ~~It~~A good agreement is~~ shown that a well coincidence has been~~ achieved, as shown in Fig. 4 (a).

$$f(R_{cur}, T_{cur}) = min\ (d) \qquad (2)$$

~~It is shown that the~~A falcon can coordinate its wing shape passively using a suite of unique mechanisms~~,~~; thus, wing skeleton motions of the carcasses represent~~ed~~ a total~~ly~~ 3D transformation rather than in a 2D plane, as shown in Fig. 4 (b~~-~~–d). ~~It~~This is irrefutably pro~~of~~ve~~d~~n by the ulna and radius, which form the skeleton of the antebrachium~~ like~~, as in other mammals.

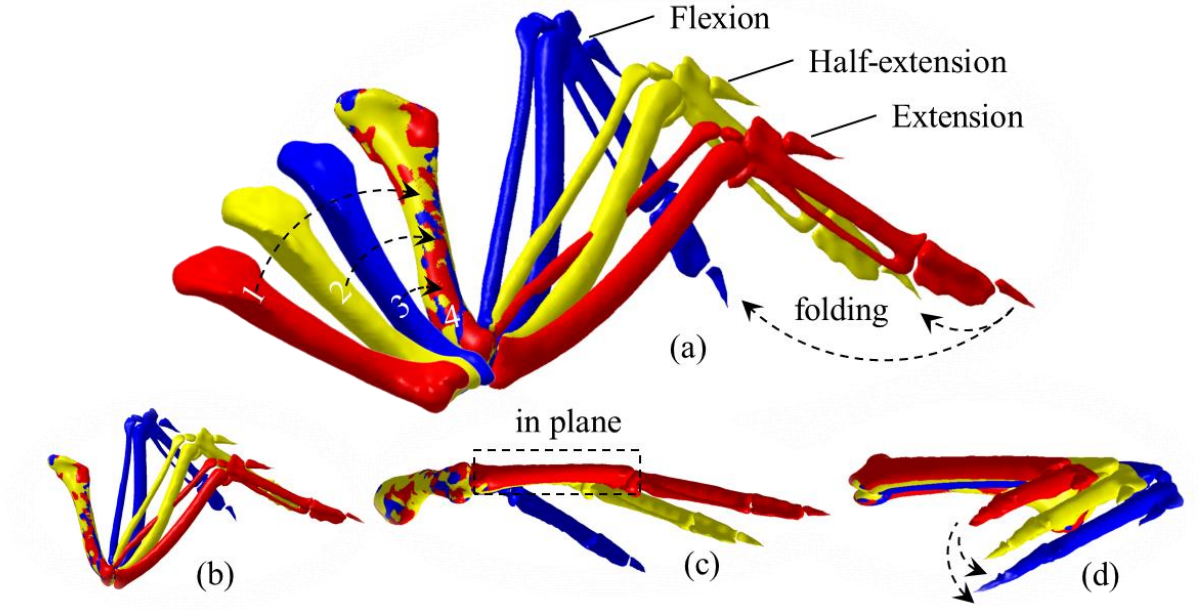

Fig. 4 Point cloud of the scanned wing skeletons: (a) illustrat~~e~~ion ~~how the~~of three humerus' (1–3) ~~were~~ transform~~ed~~ation to the exact position 4 using the proposed optimization method, and the combined skeletons at extension, half-extension, and flexion status; (b–d) dorsal, back and right view of the three skeletons.

To quantify the full range of the wing motions, a simplified seven-bar skeletal model of the falcon wing, including four main wing bones (humerus, ulna, radius, metacarpus), two wrist bones (ulnare~~,~~ and radiale) [34] and a digit (phalanx digit II~~)~~), was used to represent the 3D skeletal kinematics, as shown in Fig. 5 (a);~~.~~ ~~While the~~t~~T~~he distal phalanx digits I and III were simplified. ~~But motions of~~ However, the radiale and ulnare motions were not measured in the current ~~research for that~~study because these bones are too small to reconstruct at the adopted precision of 0.~~625mm. Considering that the~~625 mm. The wing postures were manually adjusted ~~manually under~~ according to the suggestion of Pro. Fan team. ~~a~~According to their empirical experiences on bird flights, each wing was ~~adjusted~~ scrupulously adjusted and scanned thereafter ~~scanned~~. However, these wing postures were unrepeatable~~, as a result~~; consequently, no repeatability verification was discussed in the current ~~research~~study. The motions (both axis and angular) of the radius and ulna relative to the humerus, carpometacarpus relative to the ulna, and proximal phalanx digit II relative to the carpometacarpus~~, have been~~ were measured by a step-by-step point cloud fitting approach, as shown in Fig. 5 (b~~-~~–e). At the half extension position, ~~the~~a maximum rotat~~e~~ion angle of 23.44° was found for the metacarpal~~,~~; ~~on the~~in contrast~~ary~~, only ~~about~~approximately 17° was found for ~~both~~ the ulna and ~~the~~ radius. A 3D motion of the wing skeleton was ~~apparently proofed~~proven by the rotating axes that deviated from the *z*~~-~~-axis, ~~which was~~ consistent with the 3D folding motions depicted in Fig. 4 (d). ~~On the other hand~~In contrast, a relatively simple motion of proximal digit II was estimated ~~due to~~because of its ~~independency~~

~~to~~independence from the four main wing bones. Generally, all the rotating angles from extension to extraction are approximately twice the rotation~~g~~ angle from extension to half-extension, as listed in table 1.

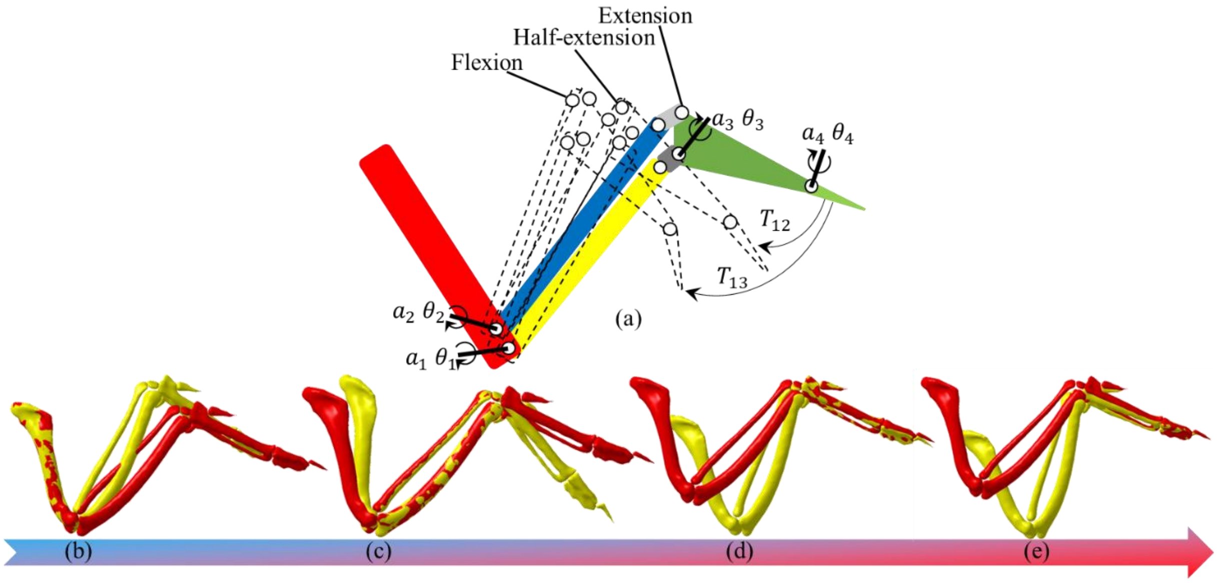

Fig. 5 ~~A~~ S~~s~~even-bar skeletal model of the falcon wing and (a)~~ and~~ the motion measurement procedures using a point cloud optimization approach (b~~-~~–e).

Table 1 Measurements of the wing skeleton during the folding

S~~s~~keleton		Extension-Half extension (°)				Extension-Flexion (°)				Axis
O~~o~~rder	B~~b~~one	Axis			Theta	Axis			Theta	deviation
1	U~~u~~lna	-0.21	-0.17	0.96	16.42	-0.31	-0.13	0.94	38.83	11.02%
2	R~~r~~adius	-0.33	-0.31	0.89	17.52	-0.33	-0.19	0.93	38.89	12.09%
3	M~~m~~etacarpal	0.28	-0.22	0.93	23.44	0.22	-0.11	0.97	55.04	12.66%
4	P~~p~~roximal digit II	-0.01	0.18	0.98	4.84	-0.12	0.39	0.91	10.02	25.32%

4 Bionic design and manufacturing

4.1 Optimization of the mechanical joint locations and rotations

In this section, we attempt~~ed~~ to replicate the wing motions ~~with~~using a fabricated mechanism. ~~Actually,~~The multi-bar mechanism paradigm inferred from anatomical studies ~~can be~~ originated ~~from~~in 1839, and a six-bar model has recently been optimi~~z~~sed with ~~a~~ high fidelity ~~recently~~ [20]. However, the previous planar four-bar mechanism did n~~'~~ot represent measured skeletal motions ~~due~~owing to its 3D characteristics, whereas the six-bar model was too complex to control for its multiple DOFs and ~~plenty of~~numerous spherical joints [35]. As a compromise, we hypothesi~~z~~sed the following possible joint types between the four ~~main~~prominent bones: a revolute joint (1 DOF) connects the distal end of the humerus with the proximal end of the ulna, a spherical joint (3 DOF) connects the distal end of the humerus with the proximal end of the radius, a revolute joint (1 DOF) connects the distal end of the ulna with the proximal end of the metacarpus, and a universal joint (2 DOF) connects the distal end of the radius with the proximal end of metacarpus, as shown in Fig. 6. These joints and bones ~~comprised the~~comprise a bionic wing skeleton with one DOF. The best~~-~~-fit axes of each joint were also optimi~~z~~sed using the global optimi~~z~~sation method *MultiStart* in MATLAB software. The cent~~er~~re locations (C) of each joint, as well as the rotation axis of the two revolute joints (A), were optimi~~z~~sed using the following equations:

~~$$\begin{cases} f(A,C) = \min(resid) \\ resid = \sum_{j=1}^{2}\sum_{i=1}^{3}\sqrt{(\vartheta_{mech}^{ij} - \vartheta_{Falcon}^{ij})^2} \end{cases} \quad (3)$$~~

~~Where superject~~
$$\begin{cases} f(A,C) = \min(resid), \\ resid = \sum_{j=1}^{2}\sum_{i=1}^{3}\sqrt{(\vartheta_{mech}^{ij} - \vartheta_{Falcon}^{ij})^2}, \end{cases} \quad (3)$$

where subject i denotes the ith angle ϑ listed in ~~Table1~~Table 1, j~~=~~ = 1 denotes the folding procedure from extension to half-extension, and j~~=~~ = 2 denotes the procedure from extension to flexion.

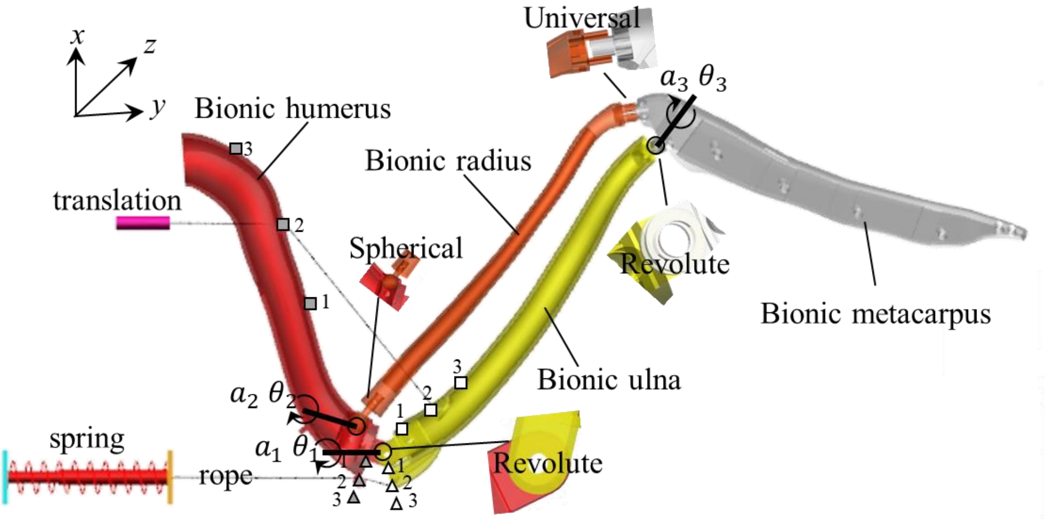

Fig. 6 Bionic wing skeleton with four ~~main~~prominent bones, four joints, ropes, and motor.

~~Elbow~~The elbow joint plays a ~~key point~~crucial role in ~~the~~ wing extending/folding~~,~~; therefore, revolute joints were used to connect the humerus-ulna~~,~~ and ulna-metacarpus~~. Due~~ joints. ~~Owing~~Due to ~~twist~~the twisting motions ~~when extending,~~ during extension, a spherical joint aligned with a universal joint ~~were~~was used to coordinate th~~i~~ese 3D rotations. Comparisons between the bionic design mechanism and ~~the~~ falcon bones ~~were depicted~~ are shown in Fig. 7. It is shown that a high fidelity ~~has been~~was achieved considering that the maximum angle of deviation was less than 1.8° ~~of~~for all ~~the~~ joints, as listed in Table 2.

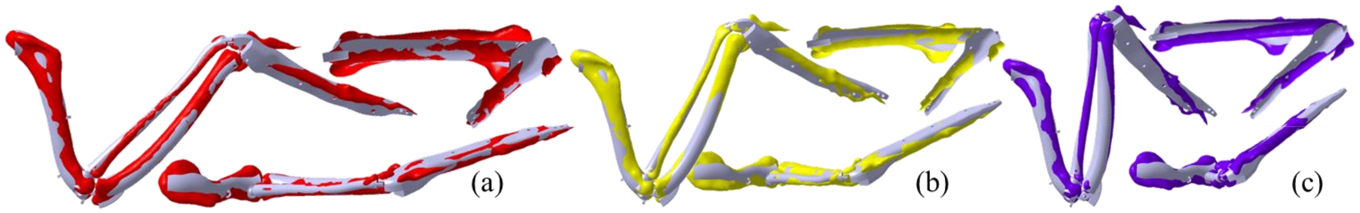

Fig. 7 Comparison of the morphing shapes between the scanned bones of a falcon and the bionic design mechanism.

Table 2 Comparison of three main joint rotations between the falcon carcass and bionic mechanical

Order	Bionic bone	Extension-Half extension (°)			Extension-Flexion (°)		
		Falcon	Mechanical	Angle deviation	Falcon	Mechanical	Angle deviation
1	U~~u~~lna	16.42	15.54	-0.88	38.83	37.18	-1.65
2	R~~r~~adius	15.72	17.52	1.~~8~~80	37.58	38.89	1.31

3	~~M~~metacarpal	23.44	22.83	-0.61	55.04	55.65	0.61

Before manufacturing, the forces loaded on the biceps brachii and ~~the~~ triceps brachii should be checked carefully to ensure the dynamic performance~~s~~ of both the motor and spring. Thus, three anchor points (represented ~~with~~by —■— in Fig. 6) were arranged on the humerus to estimate the mechanical performance~~s~~ at the proximal end of the biceps brachii, and three anchor points (represented ~~with~~by □) were arranged on the ulna to study the mechanical performance~~s~~ at the distal end of the biceps brachii. Similarly, three anchor points (▲) and three anchor points (△) were used to study the mechanical performances of triceps brachii. The quasi-steady multi-body dynamic performances were computed using the ADAMS software. Both the maximum forces and ~~the maximum~~ displacements were found when the bionic skeleton was fully folded, ~~which were~~as illustrated in Fig. 8. Considering that a large tension or displacement will decrease the dynamic performance of the micro linear actuator, ~~thus~~ a maximum force threshold ~~with 5N~~of 5 N and ~~a~~ displacement threshold ~~with a range~~ranging from ~~10mm to 20mm~~10–20 mm were used as critical values to ~~choose and~~ select motors and springs. Finally, ~~arrangement of~~ arrangements II-ii ~~&~~and B-b ~~was~~were adopted in the current research.

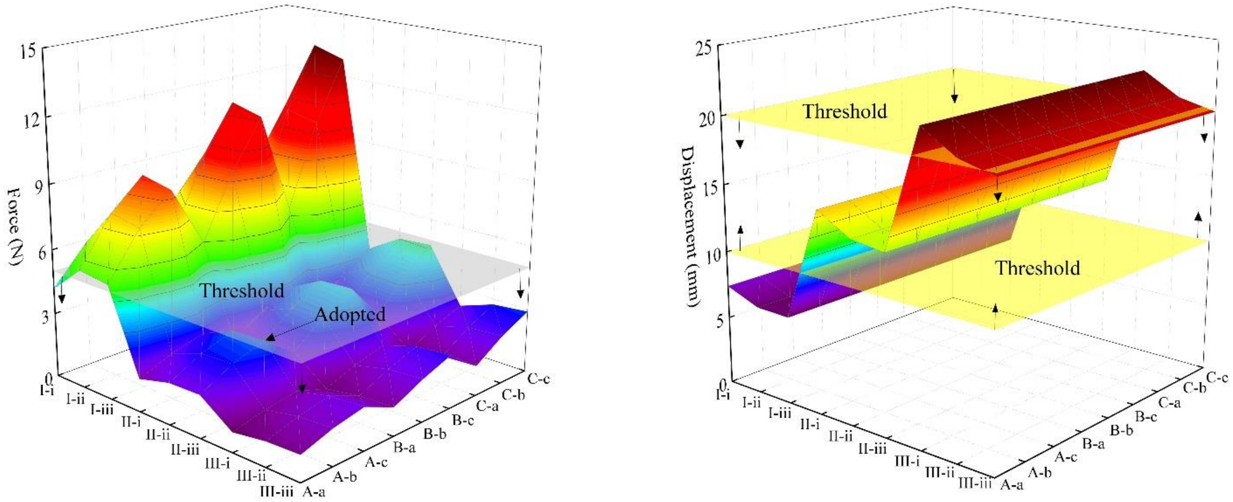

Fig. 8 Compar~~e~~ison of forces and displacements of the bionic wing skeleton during folding: I-III (■), proximal end of the biceps brachii; i-iii (□), distal end of the biceps brachii; A-C (▲), proximal end of the triceps brachii; a-c (△), distal end of the triceps brachii.

4.2 Bionic manufacturing and experimental tests

A simpler 3D four-bar mechanism with four revolute joints ~~is unable to~~cannot move along the bone motion paths, and a four-bar mechanism with four spherical joints ~~was~~is recommended to track~~s~~ the measured skeletal kinematics. A compromise simulated result was achieved, though ~~was~~ less accurate ~~then~~than that of the six-bar model [20]. However, 6 ~~degrees of freedom~~ DOF require at least 12 wires to sustain ~~its~~their morphing shape, which is too complex to control. To achieve ~~an~~ easy but stable control, a mechanism with the proposed spherical-universal-revolute joints ~~were~~was manufactured to verify the bionic design, as shown in Fig. 9.

The four ~~main~~prominent bionic bones were printed on a ~~L~~leader 3D printer (0.~~05mm~~05 mm precision, L8-410, 10~~-150mm~~–150 mm/S, Leader 3D, China). The size of the printer's build plate ~~size of the printer~~ was 410 mm × 410 mm × 500 mm. All bones were printed with a 1.75 mm layer thickness out of ~~poly~~

~~lactic~~polylactic acid material (PLA plus, density 1.~~23g~~23 g/cm3, and tensile strength 63~~-74MPa~~–74 MPa), ~~which are~~is resistant to common disinfectants and environmentally stable ~~over time~~ [36–38]. ~~To support the bones for testing, a~~A micro linear actuator container and IMU supporters were ~~also~~ printed~~. After printing, all~~ to support the bones for testing. All the printed parts were adjusted and connected using spherical~~ joint~~, universal~~ joint~~, and revolute joints, ~~which were~~ designed and manufactured with steel. ~~During adjustment, the~~ The critical length between ~~neighboring~~the neighbouring joints was measured to eliminate ~~the~~ misalignment~~.~~ during adjustment. Protective earthing (PE) wires were used to mimic both biceps brachii and triceps brachii, and they established a stable connection between the humerus and ulna to form a pair of antagonisms. During tests, the micro linear actuator (LA16-021D/P, maximum pull ~~70N~~70 N, precision 0.~~03mm~~03 mm, Inspire-robots, China) drove the bones to move through the PE wires. ~~And a~~An MCU (STM32F429) was used to control the wing skeleton motion in an open-loop ~~way~~manner that modelled the coupled motion between the elbow and wrist~~,~~. ~~t~~The wing in its extended position was referenced to measure the rotational angles during folding based on the motion capture data (MPU9250).

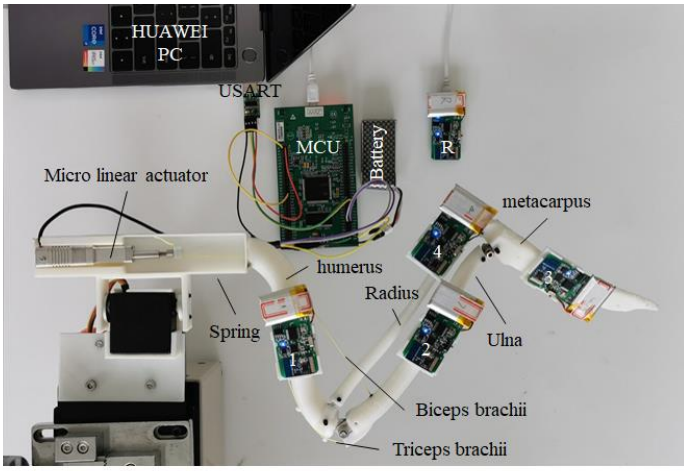

Fig. 9 ~~The~~ F~~f~~abricated wing skeleton and the motion capture system to measure folding motions.

The rotating angles of each bone were considered ~~to be the~~ critical values~~,~~; thus, the measured angles of the fabricated wing skeleton (prefix, Exp_Ang) ~~we~~are illustrated ~~as shown~~ in ~~the~~ Fig. 10. A linear control strategy of ~~4mm~~4 mm/s was used to drive the micro linear actuator (Exp_Len_Motor). It is shown that the rotating angles of the fabricated metacarpal are consistent ~~well~~ with the predicted values (prefix, Pre_Ang) in the mechanical design discussed in ~~s~~Section 4.1, with a maximum deviation of 0.59°, ~~while~~whereas a larger angle deviation was found at 1.91° for the ulna and radius. Angle deviations of the simplified 4-bar model indicate~~s~~ that radiale and ulnare may be necessary to model wing morphing accurately. Above all, the simplified 4-bar model ~~has~~ achieved a high resolution to replicate the wing folding motions by ~~optimizing~~directly optimising their joint locations and types~~ directly~~. Combined with its simplicity and robustness~~ of the mechanism~~, we estimate ~~it~~this is an enlightening bionic inspiration for morphing aircraft design.

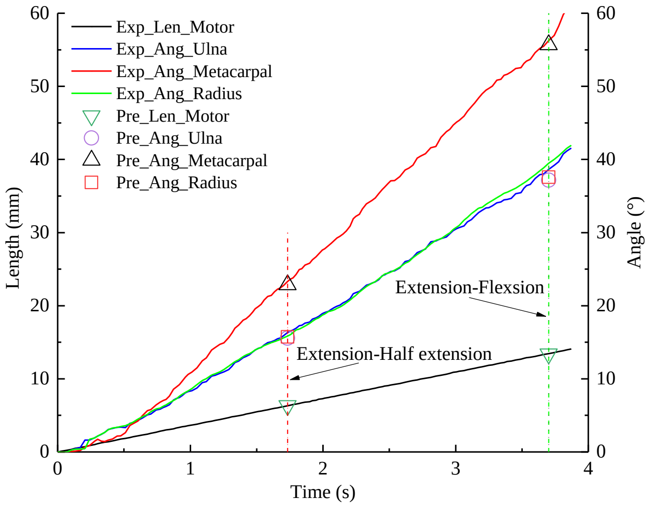

Fig. 10 Measured rotational angles of the three ~~main~~prominent bones and a comparison to the predicted data.

5 Conclusion

~~Functional~~The functional properties of muscles and ~~its~~their intrinsic mechanics and kinematics of flight have been widely investigated~~, however,~~. However, it was accomplished without ~~being able to consult~~consulting a scanned database for raptors ~~since~~because such a database was either quite incomplete to use or even ~~nonexistent~~non-existent. Particularly, the wing skeleton database of Falco peregrinus has not been examined yet. Therefore, to provide a reference for future comparative and functional studies, the forelimb of a bird of the prey *~~F~~falcon peregrinus* was studied by imaging the wings with different postures. The wing skeleton of the bird was consecutively CT-~~S~~scanned from folded posture to fully extended posture ~~consecutively, the behavior~~, and the behaviour and contribution of the four ~~main~~prominent bones during the process of folding to unfolding were ~~figured out~~determined. Finally, a bionic wing mechanism with a simple four-bar linkage ~~was~~is designed and fabricated to implement ~~the~~ folding/unfolding motions with ~~a~~ high fidelity. In this ~~paper~~study, we ~~evaluate that~~evaluated the following:

1. A carbon-fib~~er~~re suspension system with nylon wires (nonmetal material) was utilized ~~for fastening~~to fasten and ~~keeping~~maintain the hanging specimen ~~aimed at mimicking~~to mimic the motion of ~~the~~ a bird wing. The suspension system is quite suitable for bird morphing posture studies, such as CT scans, 3D shape-scans, motion ~~captures, et al~~capture, etc.

2. ~~To analyzse the kinematics of the wing quantitatively,~~ T~~t~~he myology of the forelimb skeleton ~~has been~~was studied in the current research to analyse the kinematics of the wing quantitatively. An intrinsic coordinate system at the base of the humerus was defined, and the 3D motions of the forelimb bones were represented ~~with~~by translations and rotations under the coordinates. The falcon~~-~~ wing skeleton was examined in detail, and three critical postures were scanned during folding to obtain the CT-scanned point clouds. The ~~centers,~~centres and axes of rotation of each bone were determined to mimic ~~the~~ wing motion quantitatively using a pose optimi~~z~~sation method~~ in a quantitatively way.~~. Thereafter, the kinematics of the ~~wing can be analyzed~~ wings were quantitatively analysed.

3. The ~~10~~ten forelimb bones were simplified to ~~the~~an artificial mechanism as a seven-bar model to represent ~~the~~ 3D skeletal kinematics. ~~By~~Using a step-by-step point~~-~~ cloud fitting approach, the relative axial and angular motions of the four ~~main~~prominent bones were extracted, followed ~~with the~~by quantitative measurements of the wing skeleton during the folding/unfolding motions. Inspired by these observations, a bionic mechanism with revolute, universal, and spherical joints was proposed, and the locations and rotations of each joint were optimi~~z~~sed using a global optimi~~z~~sation method to resemble the ~~reality of the~~actual motions of the forelimb.

4. Finally, a 3D four-bar mechanism (robotic-like wing) ~~was~~is fabricated ~~by~~using a 3D printer. As a good outcome, a rotational comparison of the three main joints between the falcon carcass and the artificial mechanism was ~~given~~provided. The mechanical performance~~s~~ of the artificial mechanism during folding/unfolding motions, such as maximum forces and maximum displacements, ~~were~~was computed and optimi~~z~~sed to guarantee structural integrity. ~~Also,~~ In addition, the rotational angles of the three ~~main~~prominent bones were measured and compared ~~to~~with the predicted values to show that the proposed simple four-bar model can replicate folding/~~-~~extending motions with~~ a~~ high fidelity.

The observations of the current ~~research~~study can be a useful guide for ~~morphology~~the morphological analysis of birds and an enlightening inspiration ~~to~~for the bionic design of morphing aircraft~~s~~ in the future.

References

[1] J.D. DeLaurier, An aerodynamic model for flapping-wing flight, Aeronaut. J. 97 (964) (1993) 125-130. DOI: 10.1017/S0001924000026002

[2] D. Tang, D.W. Liu, H. Zhu, et al., Shape reconstructions and morphing kinematics of an eagle during perching manoeuvres, Chinese. Phys. B. 29 (2) (2020) 024703. https://doi.org/10.1088/1674-1056/ab610a
[3] D. Tang, Z.Y. Fan, M.X. Lei, et al., A combined airfoil with secondary feather inspired by the golden eagle and its influences on the aerodynamics, Chinese. Phys. B. 28 (3) (2019) 034702. https://doi.org/10.1088/1674-1056/28/3/034702
[4] D. Lentink, U.K. Müller, E.J. Stamhuis, et al., How swifts control their glide performance with morphing wings, Nature 446 (7139) (2007) 1082-1085. https://doi.org/10.1038/nature05733
[5] D.R. Warrick, M.W. Bundle, K.P. Dial, Bird Maneuvering Flight: Blurred Bodies, Clear Heads1, Integr. Comp. Biol. 42 (1) (2002) 141-148. https://doi.org/10.1093/icb/42.1.141
[6] B.W. Tobalske, Biomechanics of bird flight, J. Exp. Biol. 210 (18) (2007) 3135-3146. https://doi.org/10.1242/jeb.000273
[7] F. Liu, J. Lin, Y. Z. Wang, et al., The Design of High Precision QCL Driver for Micro Laser Impulsed Unmanned Aerial Vehicle, Laser. J. 35 (12) (2014) 75-77. DOI: 10.14016/j.cnki.jgzz.2014.12.075
[8] J. Gerdes, A. Holness, A. Perez-Rosado, et al., Robo Raven: A Flapping-Wing Air Vehicle with Highly Compliant and Independently Controlled Wings, Soft. Robot. 1 (4) (2014) 275-288. https://doi.org/10.1089/soro.2014.0019
[9] L.Y. Matloff, E. Chang, T.J. Feo, et al., How flight feathers stick together to form a continuous morphing wing, Science 367 (2020) 293-297. https://doi.org/10.1126/science.aaz3358
[10] E. Chang, L.Y. Matloff, A.K. Stowers, et al., Soft biohybrid morphing wings with feathers underactuated by wrist and finger motion, Sci. Robot. 5 (38) (2020). https://doi.org/10.1126/scirobotics.aay1246
[11] G. Lilley, A study of the silent flight of the owl, 4th AIAA/CEAS Aeroacoustics Conference, American Institute of Aeronautics and Astronautics, Place Published, (1998). https://doi.org/10.2514/6.1998-2340
[12] J.W. Jaworski, N. Peake, Aeroacoustics of Silent Owl Flight, Annu. Rev. Fluid. Mech. 52 (1) (2020) 395-420. http://dx.doi.org/10.1146/annurev-fluid-010518-040436
[13] B.A. Mandadzhiev, M.K. Lynch, L.P. Chamorro, et al., An experimental study of an airfoil with a bio-inspired leading edge device at high angles of attack, Smart. Mater. Struct. 26 (9) (2017) 094008. https://doi.org/10.1088/1361-665X/aa7dcd
[14] C.P. van Dam, Induced-drag characteristics of crescent-moon-shaped wings, J. Aircr. 24 (2) (1987) 115-119. https://doi.org/10.2514/3.45427
[15] P. Mardanpour, E. Izadpanahi, S. Powell, et al., Inflected wings in flight: Uniform flow of stresses makes strong and light wings for stable flight, J. Theor. Biol. 508 (2021) 110452. https://doi.org/10.1016/j.jtbi.2020.110452
[16] M.Y. Huang, Optimization of flapping wing mechanism of bionic eagle, Proc. Inst. Mech. Eng. G. J. Aerosp. Eng. 233 (9) (2019) 3260-3272.https://doi.org/10.1177/0954410018794339
[17] M. Yu, P. Wu, R.B. Widelitz, et al., The morphogenesis of feathers, Nature 420 (6913) (2002) 308-312. https://doi.org/10.1038/nature01196
[18] B. Parslew, Predicting power-optimal kinematics of avian wings, J. R. Soc. Interface. 12 (102) (2015) 20140953. https://doi.org/10.1098/rsif.2014.0953
[19] K.E. Crandell, B.W. Tobalske, Aerodynamics of tip-reversal upstroke in a revolving pigeon wing, J. Exp. Biol. 214 (11) (2011) 1867-1873. https://doi.org/10.1242/jeb.051342
[20] A.K. Stowers, L.Y. Matloff, D. Lentink, How pigeons couple three-dimensional elbow and wrist motion to morph their wings, J. R. Soc. Interface. 14 (133) (2017) 20170224.

https://doi.org/10.1098/rsif.2017.0224
[21] V. Tucker, Gliding birds: The effect of variable wing span, J. Exp. Biol. 133 (1987).DOI: 10.1242/dev.02172.
[22] C.R. Lattin, M.A. Emerson, J.D. Gallezot, et al., A 3D-printed modular device for imaging the brain of small birds, J. Neurosci. Meth. 293 (2018) 183-190. https://doi.org/10.1016/j.jneumeth.2017.10.005
[23] F.D. Doty, G. Entzminger, J. Kulkarni, et al., Radio frequency coil technology for small-animal MRI, Nmr. Biomed. 20 (3) (2007) 304-325. https://doi.org/10.1002/nbm.1149
[24] S.J. Schambach, S. Bag, L. Schilling, et al., Application of micro-CT in small animal imaging, Methods 50 (1) (2010) 2-13. https://doi.org/10.1016/j.ymeth.2009.08.007
[25] M.J. Souza, J.S. Wall, A. Stuckey, et al., Static and dynamic (18)FDG-PET in normal hispaniolan Amazon parrots (Amazona ventralis), Vet. Radiol. Ultrasoun. 52 (3) (2011) 340-344. https://doi.org/10.1111/j.1740-8261.2010.01793.x
[26] T.L. Hieronymus, Flight feather attachment in rock pigeons (Columba livia): covert feathers and smooth muscle coordinate a morphing wing, J. Anat. 229 (5) (2016) 631-656. https://doi.org/10.1111/joa.12511
[27] B. Ponitz, M. Triep and C. Brücker, Aerodynamics of the Cupped Wings during Peregrine Falcon's Diving Flight, Open Journal of Fluid Dynamics, 4 (2014) 363-372. http://dx.doi.org/10.4236/ojfd.2014.44027
[28] K.E. Crandell, B.W. Tobalske, Kinematics and aerodynamics of avian upstrokes during slow flight, J. Exp. Biol. 218 (16) (2015) 2518-2527. https://doi.org/10.1242/jeb.116228
[29] K.E. Crandell, B.W. Tobalske, Aerodynamics of tip-reversal upstroke in a revolving pigeon wing, J. Exp. Biol. 214 (11) (2011) 1867-1873. https://doi.org/10.1242/jeb.051342
[30] D.B. Baier, S.M. Gatesy, K.P. Dial, Three-Dimensional, High-Resolution Skeletal Kinematics of the Avian Wing and Shoulder during Ascending Flapping Flight and Uphill Flap-Running, PLoS One 8 (5) (2013). https://doi.org/10.1371/journal.pone.0063982
[31] T.L. Hedrick, B.W. Tobalske, A.A. Blewener, How cockatiels (Nymphicus hollandicus) modulate pectoralis power output across flight speeds, J. Exp. Biol. 206 (8) (2003) 1363-1378. https://doi.org/10.1242/jeb.00272
[32] K.D. Kavanagh, O. Shoval, B.B. Winslow, et al., Developmental bias in the evolution of phalanges, P. Natl. Acad. Sci. USA. 110 (45) (2013) 18190-18195. https://doi.org/10.1073/pnas.1315213110
[33] R.A. Meyers, Morphology of the shoulder musculature of the American kestrel, Falco sparverius (Ayes), with implications for gliding flight, Zoomorphology 112 (2) (1992) 91-103. https://doi.org/10.1007/BF01673810
[34] K.R.S. Holzbaur, W.M. Murray, S.L. Delp, A Model of the Upper Extremity for Simulating Musculoskeletal Surgery and Analyzing Neuromuscular Control, Ann. Biomed. Eng. 33 (6) (2005) 829-840. https://doi.org/10.1007/s10439-005-3320-7
[35] Marghitu, B. Dan, Kinematic Chains and Machine Components Design, 2005, pp.537-581.
[36] L. Novakova-Marcincinova, J. Novak-Marcincin, Experimental Testing of Materials Used in Fused Deposition Modeling Rapid Prototyping Technology, Adv. Mat. Res. 740 (2013) 597-602. https://doi.org/10.4028/www.scientific.net/AMR.740.597
[37] K.H. Herrmann, C. Gärtner, D. Güllmar, et al., 3D printing of MRI compatible components: Why every MRI research group should have a low-budget 3D printer, Med. Eng. Phys. 36 (10) (2014) 1373-1380. https://doi.org/10.1016/j.medengphy.2014.06.008
[38] C. Chen, T. Y. Zhang, A Review of Design and Fabrication of the Bionic Flapping Wing Micro Air Vehicles, Micromachines-Basel. 10 (2) (2019) 144. https://doi.org/10.3390/mi10020144
